# A significant risk locus on 19q13 for bipolar disorder identified using a combined genome-wide linkage and copy number variation analysis

**DOI:** 10.1186/s13040-015-0076-y

**Published:** 2015-12-18

**Authors:** Magnus Lekman, Robert Karlsson, Lisette Graae, Ola Hössjer, Ingrid Kockum

**Affiliations:** Neuroimmunology Unit, Department of Clinical Neuroscience, Karolinska Institutet, Stockholm, Sweden; Department of Medical Epidemiology and Biostatistics, Karolinska Institutet, Stockholm, Sweden; Department of Neuroscience, Karolinska Institutet, Stockholm, Sweden; Department of Mathematics, Division of Mathematical Statistics, Stockholm University, SE-106 91 Stockholm, Sweden

**Keywords:** Bipolar disorder, Genome-wide, Linkage analysis, Copy number variation

## Abstract

**Background:**

The genetic background to bipolar disorder (BPD) has been attributed to different genetic and genomic risk factors. In the present study we hypothesized that inherited copy number variations (CNVs) contribute to susceptibility of BPD. We screened 637 BP-pedigrees from the NIMH Genetic Initiative and gave priority to 46 pedigrees. In this subsample we performed parametric and non-parametric genome-wide linkage analyses using ~21,000 SNP-markers. We developed an algorithm to test for linkage restricted to regions with CNVs that are shared within and across families.

**Results:**

For the combined CNV and linkage analysis, one region on 19q13 survived correction for multiple comparisons and replicates a previous BPD risk locus. The shared CNV map to the pregnancy-specific glycoprotein (*PSG*) gene, a gene-family not previously implicated in BPD etiology. Two SNPs in the shared CNV are likely transcription factor binding sites and are linked to expression of an F-box binding gene, a key regulator of neuronal pathways suggested to be involved in BPD etiology.

**Conclusions:**

Our CNV-weighted linkage approach identifies a risk locus for BPD on 19q13 and forms a useful tool to future studies to unravel part of the genetic vulnerability to BPD.

**Electronic supplementary material:**

The online version of this article (doi:10.1186/s13040-015-0076-y) contains supplementary material, which is available to authorized users.

## Background

Bipolar disorder (BPD) is a burdensome [1] and common [2] spectrum of mental disorders [3]. The concordance rate which is up to 8.5 times higher for monozygotic than dizygotic twins for BPD shows that genetic factors contribute to susceptibility to BPD [4]. Non-genetic factors are however also of importance in the underlying etiology since the heritability rates are between 59 and 87 % [4]. Accumulating data demonstrates that BPD is a both clinically and genetically heterogeneous disorder with different risk factors in different subgroups and with a shared genetic overlap between different diagnoses of psychiatric disorders [5, 6]. The emerging picture further reveals that individual genetic risk loci contribute with relatively small effect. This complicated picture has for a long time hampered the success to find robust genetic results in BPD. However, the availability to both larger sample sizes, more dense marker map [7, 8] and with application of new methodologies enforced by the venture from the Psychiatric Genomic Consortium (PGC) [9] have finally allowed for statistical robust signals from the CACNA1C, CACNB2, ODZ4, SYNE1 and NCAN genes.

The genetic architecture of BPD is however more complicated than previously anticipated [10]. A variety of genomic polymorphisms, not only restricted to variation of single nucleotides may explain why it has been hard to identify BPD susceptibility genes [11–13]. This type of variations include larger genomic segments known as copy number variation (CNV) which, in comparison to a reference genome, are defined as gain or loss of genomic segments larger than 1 kb in size [13, 14]. It was previously thought that these forms of genetic polymorphisms are relatively rare and highly potent in conferring risk [12, 15]. Recent findings indicate that besides the highly penetrant rare risk variants, common variants also occur but with a more modest risk contribution than previously assumed [12, 16]. An emerging picture thus indicates that different forms of genomic variations may explain some of the expected genetic risk for a group of individuals [10, 11, 17].

In this study our hypothesis was that CNVs irrespective of their frequencies predispose to BPD. We further hypothesized that such structural variants are inherited. Under such assumptions, families ascertained for having high genetic liability of BPD constitutes an unprecedented opportunity to find such genomic variations in regions with evidence for linkage. To increase power to find families with linkage to BPD we screened 637 BP-pedigrees, provided by the NIMH Bipolar Genetic Initiative, and selected a subsample presumed to carry a genetic form of BPD. Pedigrees were selected based on family-wise genome-wide linkage analysis or by analyzing candidate genes for presence of stretches of deletions. We selected 46 BP-pedigrees for our present study and conducted two separate forms of analyses. First, we performed parametric and non-parametric genome-wide linkage analyses using a dense SNP-marker map with genetic data filtered for genotypes mapping to CNVs.

We next tested the same sample with parametric and non-parametric linkage analyses restricted only to regions with CNVs that are shared among at least two individuals within the same family. To do this we developed an algorithm to sum family-wise linkage scores in regions with CNVs that are shared within and across families.

Our results demonstrate that for the linkage part of this study, several signals surpassed threshold of suggestive linkage for both the non-parametric and parametric models and confirm several previously reported linkage regions. For the combined CNV and linkage analysis, one region on 19q13 survived correction for multiple comparisons and confirms a previously reported risk locus for BPD.

Several plausible candidate genes for BPD reside in 19q13. Moreover, two markers in the identified CNV have been reported as eQTLs for an F-box binding protein (*FBXO30*) with a suggestive role in BPD susceptibility.

## Methods

### Study subjects and recruitment process

The BP-pedigrees and genotypic data were provided from the NIMH Bipolar Disorder Genetic Initiative [18] with ascertainment and diagnosis processes conducted during 1991 to 2001 (Wave 1–4) (detailed described in Additional file 1). This material has been analyzed for linkage to BPD in previous studies without yielding signals that meet the criterion of significant linkage (Additional file 1).

**Fig. 1:**
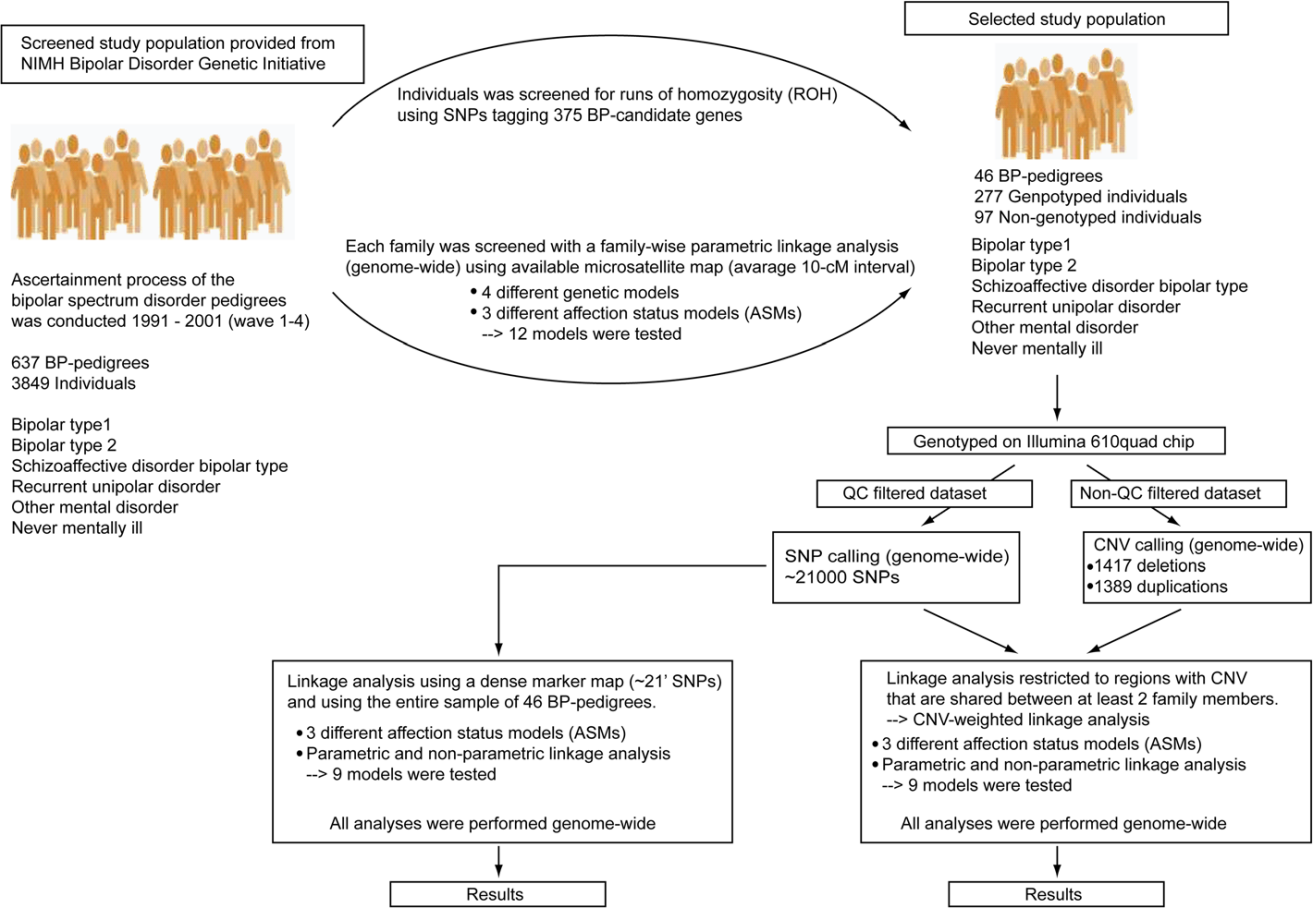
Flow chart of analytic strategy. A brief overview of our incremental strategy for finding inherited CNVs contributing to susceptibility of BPD. The flow chart illustrates our hierarchical two-stage selection procedure to reduce the entire wave 1–4 pedigree sample from NIMH Bipolar Disorder Genetic Initiative, into a smaller sample aiming to reduce heterogeneity and increase power to find segregating CNV with risk to BPD. The two screening methods we applied were a genome-wide family-wise linkage analysis and an analysis for the presence of stretches of deletions in BP-candidate genes. Calculation for linkage was performed under 3 different affection status models (ASMs). ASM 1: bipolar type 1 and schizoaffective disorder bipolar type, ASM 2: bipolar type 1 and schizoaffective disorder bipolar type and bipolar type 2, ASM 3: bipolar type 1 and schizoaffective disorder bipolar type and bipolar type 2 and recurrent depressive disorder. These analyses intend to ensure that family members were ascertained for having high genetic liability to BPD. Our selection procedure implies that a subsample of families and family members were selected out of the entire wave1–4 samples. The main features in marker calling for SNPs (using polymorphic markers) and CNVs (monomorphic markers) are shown. The flow chart illustrates the two different analyses that were used to test for inherited CNVs (i) a linkage analysis and (ii) a CNV-weighted linkage analysis which is based on our algorithm that sum the family-wise linkage scores in regions with CNVs that are shared within and across families. We addressed the issue with clinical and genetic heterogeneity for risk to BPD by categorizing individuals into 3 different ASMs and tested for parametric linkage under dominant and recessive models, and for non-parametric (HLOD) linkage

Given the complex nature of BPD we applied a screening process intended to select families presumed to carry a relatively strong genetic influence of risk to BPD (Fig. 1). In this process genotypic data from 3849 individuals in 637 nuclear and extended BP-pedigrees was downloaded from the NIMH Genetic Initiatives’ website [19] and screened with a genome-wide family-wise (defined as the type 1 error for one single family) parametric linkage scan (microsatellite map, average 10-cM interval) under different genetic models (dominant, recessive, hyper dominant or affected only) using GENEHUNTER [20] (allele frequencies and penetrance vectors described under linkage analysis).

We followed a common practice for finding linked loci of a complex disorder with an unclear classification between the different subtypes of the disorder and considered three affection status models (ASMs) [18].

The criteria we used for selecting BP-pedigrees were a family-wise logarithm of odds (LOD) score > 1.1, or if several families were found to have overlapping family-wise LOD scores > 1.0 in the same genomic region. Pedigrees were also screened for stretches of homozygous genotypes (ROH), possibly indicating deletions, as this type of genetic polymorphism has been shown to have a suggestive role in the etiology of BPD [21]. To do this, individuals in Wave1-4 BP-pedigrees were screened for deletions in SNPs tagging 357 candidate genes for BPD, by scanning for runs of homozygosity and Mendelian error analysis using the PLINK software [22]. Our ROH-based inclusion criterion was thus to find larger stretches of deletions in single individuals, and include the corresponding family regardless of whether deletions occurred in other family members.

In total we selected 46 families for our analyses consisting of 277 individuals with DNA and 97 individuals for whom DNA was not available (Additional file 2). We applied a pre-agreed analysis plan not to report a final result in the present study for a region that formed inclusion criterion for any family. In absence of interaction, this eliminates any systematic bias between the removed and retained regions. Table 1 summarizes the pedigree structure for the selected sample (Additional file 3 provides full details of the 46 pedigrees).

**Table 1:** Pedigree summary statistics and hierarchical affection status models

Pedigree structure			
Families	46		
Founders	147		
Non-founders	219		
Total number of individuals	366		
Number of affected individuals	176		
Number of BP-I individuals	168		
Number of BP-II individuals	9		
Number of SABP individuals	9		
Number of RUDD individuals	12		
Family size (average)	7.96		
Generations (average)	2.74		
Number of affected relative pairs	ASM1	ASM2	ASM3
1 degree	224	183	200
2 degree	106	99	91
3 degree	33	27	34
4 degree	6	8	10
5 degree	4	3	3
6 degree	2	2	2
Hierarchical affection status models (ASM)			
Very narrow affection status model (ASM1)	BP-I and SABP		
Narrow affection status model (ASM2)	BP-I and SABP and BP-II		
Broad affection status model (ASM3)	BP-I and SABP and BP-II and RUDD		

The table displays the summary statistics for the 46 pedigrees under the three different affection status models (ASMs). BP-I: Bipolar type 1, BP-II: Bipolar type 2, SABP: Schizoaffective bipolar type, RUDD: Recurrent unipolar depressive disorder. Individuals with a diagnosis of bipolar spectrum disorders that only apply to a certain ASM were coded as “unknown” under the other ASMs

Given the complex background of BPD, with clinical and genetic heterogeneities, and the additional uncertainty in diagnosis classification, we prioritized a smaller sample rather than a large collection of extended BP-pedigrees in the NIMH Bipolar Disorder Genetic Initiative wave 1–4. The complex pattern of inheritance in extended pedigrees complicates detection of segregating risk loci with linkage analysis. In the present linkage study, we therefore aimed to increase power using one family based criterion that tends to select pedigrees that are genetically homogeneous, and one ROH criterion which allows several smaller pedigrees to be chosen.

### Genotyping and quality controls

For the final selected individuals DNA samples (lymphoblastoid cells) were obtained from Rutgers University and Cell Repository (New Jersey, USA) and were genotyped using the Illumina Human 610quad chip at Uppsala University, Sweden. We applied quality control (QC) analysis of individuals, SNP and CNV data consisting of an ordered series of steps to prevent spurious signals that may otherwise mislead statistical inference. Of notice, in order to reduce the presence of erroneous genotypes, SNPs located within CNV-regions were removed. Methods of genotyping and QC analysis are described in Additional file 1.

After final QC filter 269 individuals with DNA from 46 pedigrees (44 pedigrees with Caucasian ancestry and 2 pedigrees with African American ancestry) and 20,714 SNP-markers were ready for the linkage analysis. CNV calling with PennCNV and QC analysis, as described in the Additional file 1, identified 2806 CNVs (1417 deletions and 1389 duplications). The mean CNV length was 110,455 bp with a maximum and minimum length of 4,580,011 bp and 10,046 bp respectively.

All of our CNVs overlapped with variations reported in three publicly available databases in August 2013; Database of Genomic Variants [23], Welcome Trust Sanger Institute [24] and finally from Children’s Hospital of Philadelphia [25].

### Linkage analysis

To unravel a segregating risk locus to BPD in our sample the different levels of heterogeneity, both clinical and genetic, prompted us to categorize individuals in different affection status models (ASMs) and to calculate linkage using multipoint parametric dominant and recessive models that take into account inter-familial heterogeneity using heterogeneity LOD scores (HLOD) and multipoint non-parametric models in MERLIN (v.1.1.2) [26].

For the non-parametric analysis we used the linear model [27] with an ‘all affected relative pairs’ (NPL_ALL_) statistics, in order to identify linkage. The Z scores were converted to LOD scores and *P* values according to Kong and Cox 1997 [20]. For the parametric dominant model we assumed a risk allele frequency of 0.0045 with penetrance vector 0.001, 0.50 and 0.75 for the three different genotypes, and for the recessive model we assumed a risk allele frequency of 0.065 with a penetrance vector of 0.0015, 0.0015 and 0.50 [28]. The GENEHUNTER software [20] was used to generate phased haplotypes and positions of recombinants.

### Empirical significance levels for linkage analysis

For the linkage part of this study we defined the suggestive linkage level to be that which on average would be exceeded by one linkage peak by chance in a genome-wide scan, and significant linkage to be that which would be expected to exceed once per 20 genome-wide scans as proposed by Lander and Kruglyak 1995 [29]. To define these thresholds we simulated 1000 datasets using the MERLIN software in which phenotypic status and pedigree structure were retained while simulating random multilocus genotypes. More specifically, these simulations are based on gene dropping in all pedigrees under the null hypothesis of no linkage. For each pedigree, marker alleles are first simulated for founders (according to their allele frequencies), then haplotypes are propagated to all non-founders based on Mendelian segregation and recombinations.

The threshold for the empirical *P* value corresponding to suggestive linkage was then calculated based on a family-wise error rate (FWER) of 1-e^{−1} = 0.632, this is the probability that a Poisson distributed random variable with an expected value of 1, is positive, and it approximates the probability of at least one significant linkage peak [29].

As the models are nested, correction for multiple comparisons across the different models would have been too conservative, we corrected only within each model. Additional file 4 reaffirms that the linkage peaks of all nine models are strongly correlated.

### Algorithm of CNV-weighted linkage scores

In order to find inherited genomic regions conferring risk for BPD, the sum of average family-wise parametric LOD scores or non-parametric Z scores were calculated over regions and families sharing overlapping CNVs. For families with at least two members with overlapping CNVs, the average linkage score in the region was calculated and added to those observed in the same region in other families. Thus, the algorithm generates a CNV-weighted linkage scores for genomic segments representing regions with CNVs that are shared within and across families (detailed described in Additional file 1 and Additional file 5).

### Empirical significance levels for CNV-weighted linkage scores

**Fig. 2:**
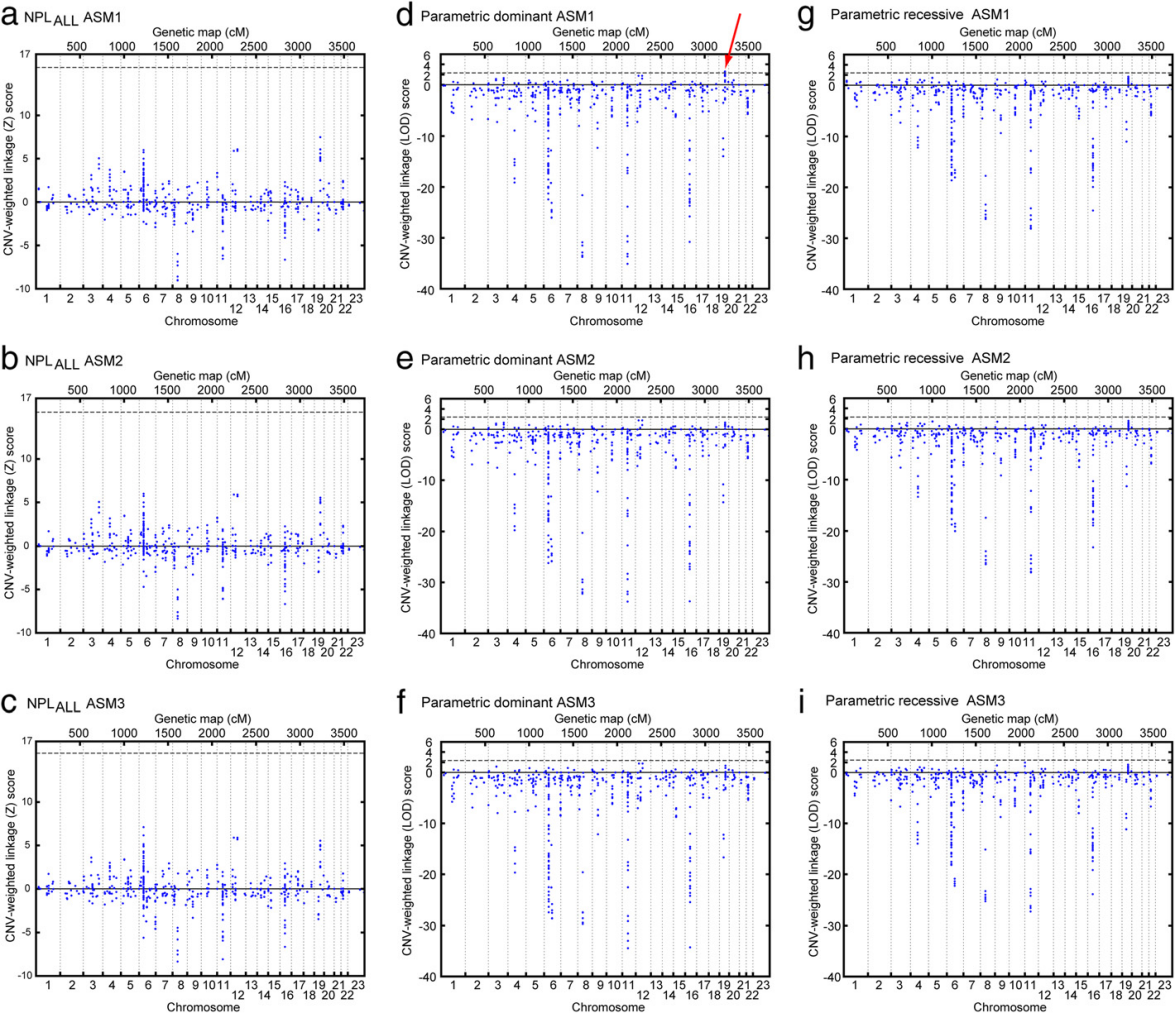
Results of the genome-wide CNV-weighted linkage analyses. The plots illustrate genome-wide CNV-weighted linkage scores of the parametric (dominant and recessive) and non-parametric (NPL_ALL_ statistics) models under the three affection status models (ASM1-3). The sum of average family-wise linkage scores LOD scores for parametric and Z scores for non-parametric models were calculated over regions with the presence of copy number variation that is shared between at least two individuals within and across families. The thresholds for significance (dotted lines) were defined after a 1000-fold simulation analysis including FWER correction

Empirical *P* values for the CNV-weighted linkage score analyses were also derived by gene dropping from simulated multilocus genotypes in MERLIN, using the same marker allele frequencies of the founders as in the unweighted linkage analysis simulations. For these analyses the threshold for significance was defined based on a FWER of alpha = 0.05. The nested models made us to correct within each model and not across the different models. Figure 2 reaffirms that the CNV-weighted linkage peaks of all nine models are strongly correlated.

## Results

### Linkage analysis

The multipoint non-parametric (NPL_ALL_) linkage analysis met suggestive level on 3p14.1. The three affection status models generated comparable results with ASM3 exhibiting the strongest peak, NPL Z = 3.56 [20] (Tables 2 and 3 and Additional file 4). With the parametric dominant model suggestive linkage, HLOD = 2.41, was reached for the same region (3p14.1) as in the non-parametric model (Table 2 and 3 and Additional file 4). For the recessive model, the most notable evidence of linkage occurred at 6p12.3 (HLOD = 2.64). Other regions reaching suggestive linkage were 1q23.3 (HLOD = 2.04) and 10q26.2 (HLOD = 2.25), all with ASM3.

**Table 2:** Summary table of the suggestive linkage results

Maximum linkage scores of parametric linkage analysis									
Chr	95 % C.I. (cM)	Peak marker	Locus	HLOD (threshold)	Alpha	IC	Diagnostic model	Genetic model	Empirical *P* value (threshold)
1	6.2	rs10919096	1q23.3	2.04 (1.97)	0.20	0.95	ASM3	Recessive	0.57 (0.632)
3	4.9	rs1001763	3p14.1	2.41 (1.97)	0.29	0.91	ASM3	Dominant	0.32 (0.632)
6	5.2	rs9381631	6p12.3	2.64 (1.97)	0.28	0.91	ASM3	Recessive	0.19 (0.632)
10	4.5	rs638395	10q26.2	2.25 (1.97)	0.28	0.93	ASM3	Recessive	0.40 (0.632)

The table displays maximum linkage scores for the suggestive linkage results of the non-parametric and parametric linkage analyses

A 1000-fold simulation analysis generated genome-wide thresholds for suggestive linkage Z and parametric HLOD scores (shown in parentheses). The approximate 95 % confidence intervals for the highest linkage scores are defined as a LOD-drop, or Z-drop, corresponding to a unit of 1.0. The mean information content (IC) estimate across all chromosomes was 0.92 (range 0.96–0.70), indicating that most of the genetic information was successfully captured using our high density mapping approach. The fraction of linked families, alpha, was less than 0.29 for regions showing suggestive linkage, indicating that there is evidence for a marked heterogeneity with respect to linked loci in these 46 pedigrees

*Abbreviations: NPL Z* the linkage statistics for the estimation of *identity-by-descent* (IBD) allele sharing. *LOD* is the LOD calculated from NPL Z sores according to Kong and Cox (1997). *IC* the information content and is a measure of the probability that the IBD status at a certain locus can be determined for a given pairs of relatives. *Alpha* a measure of the locus heterogeneity that indicates the proportion of families with alleles linked to disease at a certain locus. *HLOD* estimate of the heterogeneity LOD score. *Delta* measure of allele sharing among affected individuals within pedigrees. *Empirical P value* estimated probability of having a score by chance that is at least as large as the observed one, after correction for multiple comparisons

**Table 3:** Summary table of the suggestive linkage results

Maximum linkage scores of non-parametric linkage analysis									
Chr	95 % C.I. (cM)	Peak marker	Locus	NPL Z (threshold)	LOD	IC	Delta	Diagnostic model	Empirical *P* value (threshold)
3	18.5	rs4855407	3p14.1	3.56 (3.34)	2.34	0.91	0.50	ASM3	0.46 (0.63)
3	3.49	rs4855407	3p14.1	3.49 (3.32)	2.32	0.91	0.49	ASM2	0.48 (0.63)
3	18.5	rs4855407	3p14.1	3.56 (3.30)	2.34	0.91	0.43	ASM1	0.41 (0.63)

The table displays maximum linkage scores for the suggestive linkage results of the non-parametric and parametric linkage analyses

A 1000-fold simulation analysis generated genome-wide thresholds for suggestive linkage Z and parametric HLOD scores (shown in parentheses). The approximate 95 % confidence intervals for the highest linkage scores are defined as a LOD-drop, or Z-drop, corresponding to a unit of 1.0. The mean information content (IC) estimate across all chromosomes was 0.92 (range 0.96–0.70), indicating that most of the genetic information was successfully captured using our high density mapping approach. The fraction of linked families, alpha, was less than 0.29 for regions showing suggestive linkage, indicating that there is evidence for a marked heterogeneity with respect to linked loci in these 46 pedigrees

*Abbreviations: NPL Z* the linkage statistics for the estimation of *identity-by-descent* (IBD) allele sharing. *LOD* is the LOD calculated from NPL Z sores according to Kong and Cox (1997). *IC* the information content and is a measure of the probability that the IBD status at a certain locus can be determined for a given pairs of relatives. *Alpha* a measure of the locus heterogeneity that indicates the proportion of families with alleles linked to disease at a certain locus. *HLOD* estimate of the heterogeneity LOD score. *Delta* measure of allele sharing among affected individuals within pedigrees. *Empirical P value* estimated probability of having a score by chance that is at least as large as the observed one, after correction for multiple comparisons

### CNV-weighted linkage analysis

We identified 2806 CNVs in our families all of which overlapped with previously reported CNVs in three publicly available databases (see Materials and Methods).

**Fig. 3:**
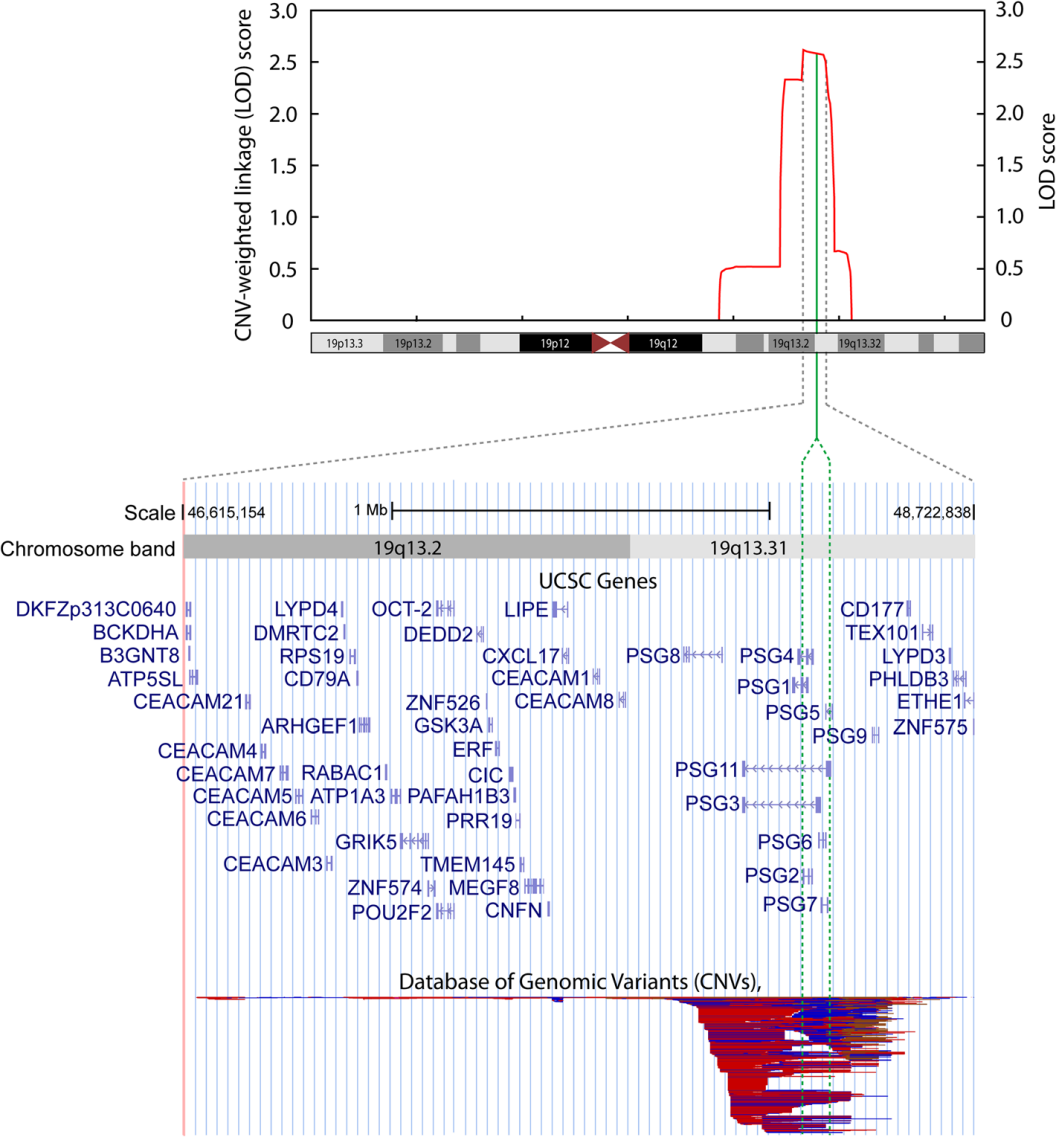
CNV-weighted linkage analysis at 19q13. Linkage scores and CNV-weighted linkage scores are illustrated relative to UCSC genes and structural variations in Data Base of Genomic Variation (DGV). The plotted red linkage curve represents results of the LOD scores from 5 pedigrees (pedigree-id: 29–0209, 29–0174, 26–5011, 20–1049 and 12–330), consisting of 12 individuals (ind-id: 29–10642, 29–10656, 29–10528, 29–10535, 29–10532, 26–50071, 26–50069, 20–10868, 20–10856, 12–11239, 12–11241 and 12–11240) who shared a CNV and which generated CNV-weighted linkage scores (chr19:48066441–48114839 and chr19:48114839–48157656) that survived correction for multiple comparisons (empirical *P* = 0.033). The green vertical line marks the location of the shared CNV from these 5 families relative to the linkage peak and relative to the UCSC genes. Lower panel displays reported structural variations from DGV. Color scheme of DGV CNVs; blue: gain, red: loss, purple: inversion, black: unknown, and brown: both loss and gain. All genomic coordinates are according to NCBI36/hg18

**Fig. 4:**
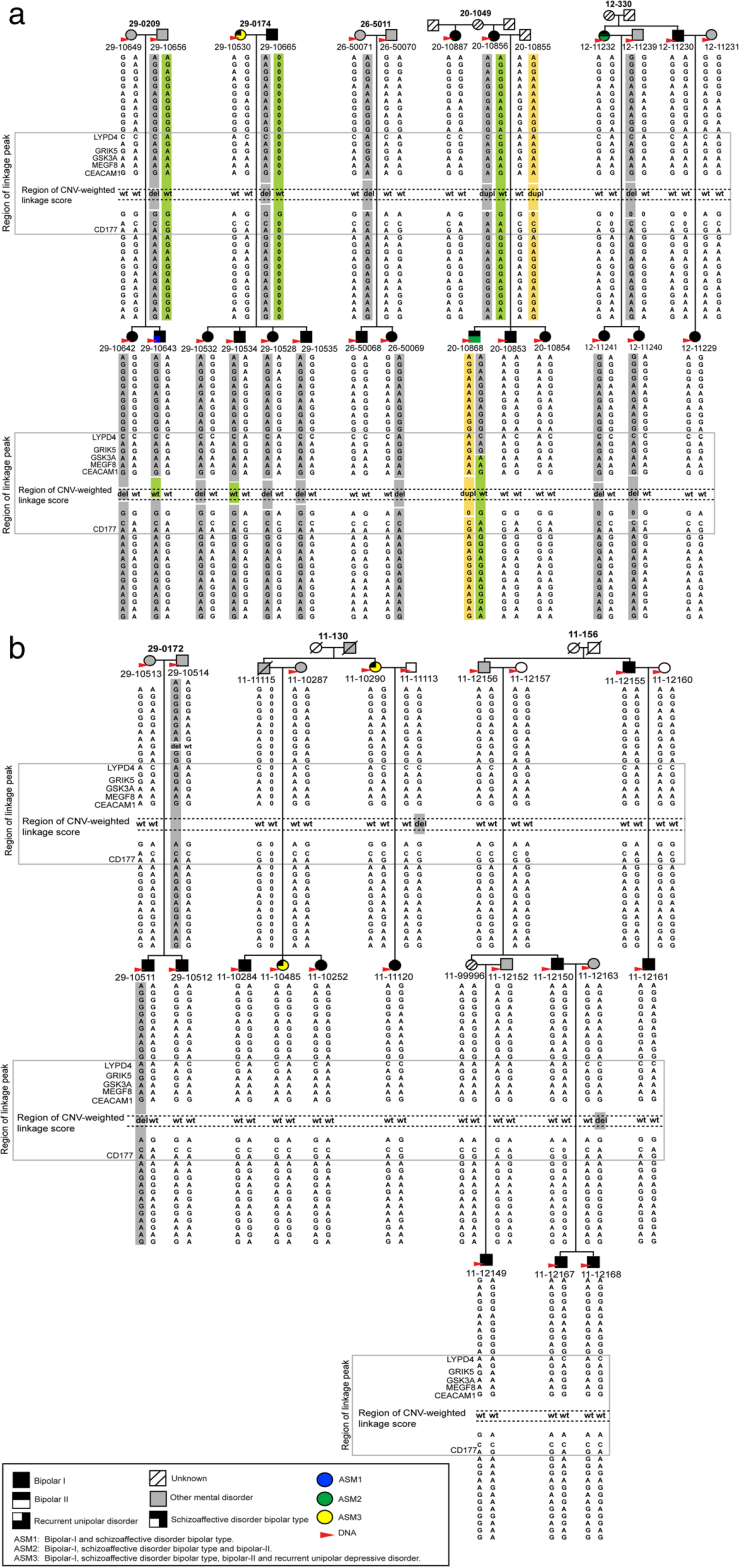
Haplotype analysis of families with CNVs in 19q13. Phasing analysis of genotypes to generate the most likely haplotype in pedigrees with CNVs on chromosome 19q13, was carried out with the GENEHUNTER software. Forty-seven markers representing all available markers in this region, spanning a region of 17.04 Mb, were included for the haplotype analysis. To simplify illustration of results, flanking markers were removed and only genotypes for 33 markers most proximal to the CNV are depicted, mapping a 6.05 Mb region. The linkage peak region is marked with a gray window and spans 1.5 Mb. The region with the two adjacent significant CNV-weighted linkage scores (91,215 bp in size) is illustrated with a gray dashed line. CNVs of duplication are denoted ‘dupl’, deletions are denoted ‘del’ and the normal state (wild type) are denoted ‘wt’. Haplotypes are displayed in colors (only for relevant chromosomes) to illustrate inheritance of gain/loss of genomic segments. The relative genomic region of each CNV is illustrated by separate colored segments. Of note, CNV calling was made based on a complete set of non-QC filtered sample of both monomorphic and polymorphic probes whereas analysis of the haplotypes was made using QC filtered polymorphic probes only. In order to retrieve recombinant mapping of high resolution, all available SNP-markers located within the linkage peak region were included. Representative gene-id’s are displayed. All genomic coordinates are according to NCBI36/hg18. **a** Results of the initial analysis of CNV-weighted linkage scores with 5 pedigrees consisting of 12 individuals with a shared CNV. In pedigree 29–0174 no DNA was available for individual 29–10665 who was therefore excluded from the initial CNV-weighted analysis. The CNV status for this individual was revealed in the subsequent phased haplotype analysis. Moreover, in pedigree 20–1049 the CNV-carriership in 20–10855 was detected using the subsequent phased haplotype analysis. **b** Results of the extended analysis to find undetected CNV’s. In our first attempt to identify undetected CNV’s in this region we manually checked the CNV calling and identified 3 individuals with deletions, 11–11113, 11–112163 and 29–10511, and individual 29–10514 with a deletion in the adjacent region. Finally, a phased haplotype analysis indicates that the CNV in 29–10511 is a *de novo* event and that no transmission of CNV’s occurs in the pedigrees 11–130 and 11–156. This analysis further indicates deletions in 29–10665 and 20–10855

Of the nine different linkage and affection status models that were tested only the parametric dominant model, under a narrow diagnose classification (ASM1), exhibited a significant CNV-weighted linkage score after a 1000-fold simulation analysis including correction for multiple comparisons, with empirical *P* = 0.033 (Fig. 2). The significant signal that exceeded the genome-wide threshold (dashed line of Panel d in Fig. 2) resides on 19q13 and represents a region of CNVs that due to our algorithm design was divided into two separate segments (chr19:48066441–48114839 and chr19:48114839–48157656) (Fig. 3) and was generated from 5 families (Fig. 4a). The positions of each CNV are provided in Additional file 6. CNVs were present among 12 individuals, 9 of which were affected under the narrow affection status model (ASM1) in the 5 families contributing to the CNV weighted linkage score (illustrated in Fig. 3).

The 19q13 region exhibits frequent CNVs in the general population and stretches over a region that harbors a family of genes encoding the pregnancy-specific glycoproteins (*PSGs*) (Fig. 3).

Since the CNV-weighted linkage score analysis did not require a positive CNV status only among those affected, according to the different affection status models, we next attempted to find the implications of structural variations in the *PSG* genes for risk to BPD. In particular, we wanted to found if the CNVs are *de novo* or inherited by performing a haplotype analysis in the families that contributed to the CNV-weighted linkage peak (Fig. 4a).

In pedigree 29–0174 we observed a deletion in three affected children, but not in the fourth affected child (29–10534) which otherwise has inherited the same haplotype as his siblings from the father for whom we did not have any DNA sample. This indicates either that there is a double crossover in this region or that we missed the detection of a CNV in the child or that the father has two identical haplotypes with the exception of the presence of the CNV.

Similarly, in pedigree 29–0209 the son (29–10643) has inherited the same haplotype as his sister from the father, but no CNV was detected despite being present in both the father and the sister. This could be explained either by a double crossover or by us failing to detect the CNV. In family 26–5011 the haplotype with the CNV was only transmitted to one of the affected children. In family 20–1049 the CNV was transmitted from the untyped father (20–10855) to a child with diagnosis under ASM2 but not to the sibling with diagnosis under ASM1.

This prompted us to identify plausible undetected CNVs, by investigating non-QC data in the entire sample in this region. We observed deletions for three individuals (11–11113, 11–12163 and 29–10511) in three additional pedigrees (11–130, 11–156 and 29–0172) (Fig. 4b). We further observed that individual 29–10514 has a deletion adjacent to the region of interest. A haplotype analysis of these three pedigrees revealed a *de novo* CNV in pedigree 20–0172 for individual 29–10511 and deletions in families 11–130 and 11–156 that were not transmitted (Fig. 4b) and therefore not included in our CNV-weighted linkage analysis. From the haplotype analysis it seems unlikely that we have missed to detect CNVs in this region in other family members of these three families.

## Discussion

### Linkage analysis

Our genome-scan using 20,123 markers resulted in a high resolution mapping to identify narrow regions linked to BPD. We performed QC analyses aiming to reduce influences from markers located in CNV regions and selected informative markers using an independent study population. We estimated thresholds for suggestive linkage through simulation according to a well established criteria of Lander and Krygluak 1995 [29] to obtain robust linkage data. Four loci reached suggestive evidence of linkage to BPD.

The most notable linkage was to chromosome 3p14.1. This signal reached suggestive linkage in both the non-parametric and parametric dominant models under ASM3, and is consistent with several previous reports of linkage to BPD [30–32]. Several candidate genes related with synaptic and other functions of relevance to BPD susceptibility reside in this region [32]. Of note, one of the suggested genes, *MAGI1*, has recently been reported [33] to harbor large structural variation (CNV) in the same BP-pedigree sample as was used in the present study. The *MAGI1* gene polymorphism was further validated in an independent sample of unrelated bipolar, schizophrenia and schizoaffective disorder cases, and thus, represents an interesting candidate for future studies. The synaptic function gene, *MAGI1*, has also been proposed as a putative candidate in a linkage and expression analysis by Lopez de Lara 2010 [32]. However, it is unlikely that the *MAGI1* CNV is the major reason for the observed linkage at 3p14 in our sample since the CNV reported by Karlsson et al. [33] was only observed in two (11–158 and 11–130) of the 46 BP-pedigrees used in both studies. This argument is supported by the notion that under the parametric linkage 29 % of the pedigrees are estimated to be linked to this region (see Table 2). The non-parametric linkage obtained without pedigree 11–158 and 11–130 was as high as 1.56, while it was 1.16 in these two families alone.

Our suggestive linkage to 1q23 in the parametric recessive analysis is consistent with the model-free linkage analyses reported previously [34, 35]. This locus has also been linked to schizophrenia [36] which further supports a shared genetic vulnerability between schizophrenia (SZ) and BPD. There have been several reports of linkage to 10q26.2 [31, 37, 38] where we detected suggestive linkage for the recessive model.

Although chromosome 6 has been a focus for a BPD risk locus [39, 40], none of these regions overlap with 6q16 reported in our study.

The present study was aimed for a CNV-weighted analysis and apparently several pedigrees in our sample were not optimal for a linkage analysis. Since the number and sizes of pedigrees were small, the number of meioses was limited, leading both to low power and reducing the ability to pinpoint linkage to a small region. Of note, the same BP-pedigrees have been included in previous linkage scans of both parametric and non-parametric models [41] without yielding suggestive linkage in regions overlapping with those identified in the present study. This notion underlines the profoundly heterogenetic background of BPD and motivates methods attempting to search for shared segregating risk loci in a more homogenous sample than in the present study.

### CNV-weighted linkage analysis

Based on 2806 CNV segments, which were all found in the general population, the parametric dominant ASM1 model identified two CNV-weighted linkage scores on 19q13 that remained significant after a 1000-fold simulation including a FWER correction (empirical *P* = 0.033). We did not adjust thresholds for significance for the CNV-weighted linkage analyses according to all tested models. Our CNV-weighted linkage score on 19q13 would not be significant if all nine models were accounted for according to Bonferroni’s approach. However, a Bonferroni correction would be too conservative as the models are correlated (see Fig. 2). Our algorithm identified 12 CNV-carriers in 5 families that contributed to this signal (Fig. 4a). The CNV polymorphism is located in a region with frequent structural variations and harbors a gene family encoding the pregnancy-specific glycoprotein (*PSG*) genes (Fig. 3).

In a series of subsequent analyses we aimed to find possible implications of structural variation in the *PSG* gene in vulnerability to BPD as well as to unravel if the CNVs are inherited or *de novo*. To do this we analyzed phased haplotypes in the 19q13 region and manually checked the CNV calling prior to QC filtering. We found that in the 5 BP-pedigrees contributing to the CNV-weighted linkage score on 19q13 under the ASM1 model, 9 of 17 were affected (BP-I) but also that 5 of 15 unaffected (i.e. not classified BP-I) individuals were positive for the CNV, that is, they had a deletion (del) or duplication (dup) of a chromosomal region that included the CNV-weighted linkage peak. This indicates that if the CNV is functional in causing risk for BPD it still has incomplete penetrance, whereas some individuals got BPD due to other reasons.

This observation can also be construed as the CNV having no involvement in the etiology of BPD. Although this cannot be entirely ruled out, the counter-argument that nonetheless underscores the involvement of the CNV polymorphism in 19q13 in the etiology of BPD concerns issues of disease classification. In spite of the fact that this polymorphism occurs in individuals without disease classification according to ASM1, they are not classified as never mentally ill. The fact that the CNV deletions and duplications are not entirely overlapping are most likely due our CNV calling algorithm based on SNPs, as described in the Additional file 1. Of importance, none of the suggestive linkage signals or significant CNV-weighted linkage scores occurred in regions in which families were selected (Additional file 2).

### Possible candidate for BPD in 19q13?

Of interest, our result with a significant signal on 19q13 for BPD susceptibility agrees with previous reports.

First, without implicating a specific gene, Francks et al. [42] detected linkage of 19q13 to both SZ and BPD. The 19q13 locus harbors several putative candidate risk genes for BPD, e.g. the glycogen synthase kinase 3-α (*GSK3A*) gene and the glutamate receptor, ionotropic kainate 5 (*GRIK5*) gene. The GSK3A protein is homologous to *GSK3B*, a target molecule for lithium treatment [43], which has regulatory functions on proteins with a reported role in BPD susceptibility [44]. Several genes pertaining to the glutamate system have consistently been associated with BPD [39]. Taken together, there is a strong support for both the glutamate and the cell growth-maintenance related genes in BPD etiology.

Secondly, Alkelai and collaborators [45] found the *CEACAM21* gene in the 19q13 region to associate with SZ. The CEACAM genes, or the carcinoembryonic antigen-related cell adhesion (*CEA*) gene family, have several structural and functional similarities to the *PSG* genes (OMIM: 109770). Recent results show that the CEA genes are both brain and immune cell expressed (http://genome.ucsc.edu/). Although the exact molecular function of these gene products remains elusive, some studies reveal function related to cell-cell adhesion, innate immune system and signal modulation in various tissues [46–48]. These lines of results, in parallel to the enrichment of immune system genes among those associated or linked to BPD [49, 50] suggest a possible role for the *PSG* gene in BPD etiology.

Other candidates in this linkage peak region include genes related to neurotrophin [39] and immune systems [51] (Fig. 3), which is interesting, given that such mechanisms have been proposed to be involved in the etiology of BPD and other psychiatric disorders.

The mechanisms of the clinical manifestations and phenotypic effects of CNVs are well documented [52] and include alteration of gene dosage, truncated protein or positional effects. The latter include a transcription that may be directly controlled by promoters in the CNV or by alteration of chromatin structure [53]. Of note, regulatory elements have been identified as far away as 2 Mb from the transcription unit [52, 53].

Based on these observations we made a bioinformatic search (http://regulome.stanford.edu/) and screened all available markers in the CNV region for being transcription factor binding sites. Two markers (rs4802370 and rs7252967) are likely transcription factor binding sites and linked to expression of the F-box binding gene (*FBXO30*). The F-box protein functions as an ubiquitin-ligase and targets the transcription factor *NF-kB* [54]. Of interest, the *NF-kB* pathway has been shown to be a key regulator of neuroplasticity, neuronal survival and pro-inflammatory status and thus serves as a one of many etiology correlates to BPD [55, 56].

In a previous study, Karlsson et al. 2012 [33], identified a rare and highly penetrant CNV which map in the MAGI1 gene. The Karlsson et al. study analyzed the identical dataset as was used in the present study and reported the MAGI1-CNV in the pedigrees 11–158 and 11–130. Although the polymorphism in the *MAGI1* gene in pedigrees 11–158 and 11–130, reported by Karlsson et al. 2012 [33], was ranked among the 25 strongest candidates in the CNV-weighted linkage analysis it was not the strongest observation (Additional file 7). This is explained by the non-overlapping CNV segments between these two families.

### Limitations

Although our approach was successful in reducing genetic heterogeneity and evaluating linkage restricted to regions with shared CNVs there are several limitations in this study.

Firstly, CNVs were called if they were longer than 10 kb. Several lines of evidence suggest that CNVs shorter than 100 kb are less consistent using SNP-array CNV calling [57]. Of note, shorter structural variations have gained a great deal of attention for their role in complex disorders [13, 58]. Thus, for the purpose of not rejecting *a priori* true positive CNV segments with a putative role for BPD-vulnerability, shorter segments were allowed in this study. The CNVs in 19q13 were all longer than 100 kb, except for one CNV of length 91,215 bp.

Secondly, properties of the algorithm and definition of shared CNVs have consequences for the final results. It can be argued that sharing of CNVs between more than two individuals in the same family should be used for identification of CNV with major implications on susceptibility for BPD. The limited pedigree size prompted us to set this threshold at two individuals.

Thirdly, the CNV-weighted LOD score method has the potential to highlight regions where presence of a segregating CNV in pedigrees correlates with a higher family-wise LOD score. Since this approach simply sums LOD scores across families with at least two CNVs, but otherwise do not weight the family LOD scores by the CNV frequencies, CNVs which are strongly correlated with high LOD scores in a small fraction of pedigrees would rank lower in comparison to what would be seen for less strongly correlated CNVs found in a large proportion of pedigrees. It would therefore be of interest in future studies to weight the family linkage scores in different ways in terms of their family members CNV. In fact, CNV-weighted linkage analysis is an instance of combined association and linkage analysis, for which different methods and score functions have been proposed, see for instance [59–61] and references herein.

Fourthly, in the present study we set the threshold for defining a shared CNV as being strict overlapping. It is unclear whether this categorization is necessary for it to contribute to increased risk for disease [12, 62, 63]. Thus, our study design may possibly have led to rejection of putative disease causing CNVs.

Lastly, although the PennCNV algorithm reports CNVs with a high power and at a low false positive rate [64], we cannot exclude that we missed to detect CNVs in our dataset.

In summary, our study provides statistical evidence that a region on 19q13 could be tied to BPD, which raises the possibility that this region confers risk to BPD for a subsample of individuals. Still, these results are as yet inconclusive for a specific candidate gene. Further studies are needed in independent samples in order to confirm the involvement of CNVs at 19q13 in BPD susceptibility and to understand the molecular consequences of such a CNV. Nonetheless, we conclude that our CNV-weighted linkage approach is a useful tool for future studies, attempting to address the role of larger structural variants in multifactorial diseases such as BPD.

## Additional files

Additional file 1: **Supplementary materials.** (DOC 43 kb)

Additional file 2: **Selection criteria for inclusion of BP-pedigrees.** Data obtained from a large number of pedigrees from NIMH Genetic Initiative Wave 1–4 was screened to generate informative pedigrees and to reduce any sporadic and environmental form of BPD. Two different analyses were used to select pedigrees. Test for runs of homozygosity (ROH) and analyzing regions with increased parametric family LOD scores under different assumed modes of inheritance. (DOC 62 kb)

Additional file 3: **Pedigrees included in linkage and CNV weighted analysis for BPD.** To retrieve informative pedigrees from the NIMH Genetic Initiative Wave 1–4 sample, 46 pedigrees consisting of 269 genotyped individuals and 97 individuals with no available DNA were selected. Three affection status models were considered (ASM1-3) based on the different bipolar affective disorder sub-types, described in the figure-box. Individuals with a diagnosis of bipolar spectrum disorders which only apply to a certain ASM were coded as “unknown” under the other ASMs. In order to illustrate full details of pedigree composition different subtypes of bipolar spectrum disorders are illustrated. For linkage analyses with different ASMs only genotyped individuals were coded as ASM1-3. All other individuals, irrespective of diagnosis, were coded as ‘unknown’. (PDF 364 kb)

Additional file 4: **Results of the parametric and non-parametric genome-wide linkage analyses under affection status models ASM1-3.** Simulation based thresholds for genome-wide significant and suggestive linkage levels are illustrated in red and black dashed lines respectively. A-C: Non-parametric linkage analyses. NPL Z scores are illustrated. D-F: Parametric dominant linkage analyses. Heterogeneity LOD scores (HLOD) are illustrated. G-I: Parametric recessive linkage analyses. Heterogeneity LOD scores (HLOD) are illustrated. (PDF 367 kb)

Additional file 5: **Principles for region for which one CNV-weighted linkage score is calculated.** LOD scores from two families (family ID: 20–1049 and 12–330) for a certain chromosome are depicted. The occurrence of CNVs for individuals within these families is also illustrated. The average linkage score (parametric LOD score or non-parametric Z-score) is calculated over the region with overlapping CNV from at least two individuals in the same family. Note, for those individuals with the presence of a CNV were SNPs zeroed out. In the non-CNV carriers were genotypes intact. The average linkage score from all families with overlapping CNV in at least two individuals is added. A CNV-weighted linkage score is thus generated for a defined region that share overlapping CNVs for more than 1 individual per family. To illustrate the calculation; an example is given with three markers that are located in a region with four overlapping CNVs, at the position 60–75 cM. For these 3 markers, the LOD scores for the two families are: Fam-ID 12–330: 0.9, 0.9 and 0.9, Fam-ID: 20–1049: 0.2, 0.4 and 0.1. The CNV-weighted linkage score (the sum of average linkage score) is then calculated as: Fam-ID 12–330: (0.9 + 0.9 + 0.9)/3 = 0.9, Fam-ID: 20–1049: (0.2 + 0.4 + 0.1)/3 = 0.23. Then, a total CNV-weighted linkage score = 0.9 + 0.23 = 1.13. (PDF 179 kb)

Additional file 6: **Genomic positions of CNVs identified in 19q13.** The table shows the positions of all CNVs that were identified in 19q13 (chr19:48066441–48157656) and that harbors a significant CNV-weighted linkage score. All genomic coordinates are according to NCBI36/hg18. (DOC 38 kb)

Additional file 7: **Table of the 25 strongest CNV-weighted linkage scores of the parametric dominant ASM1 model.** The two strongest signals withstood test for significance using a 1000-fold simulation analysis including a FWER correction. Asterisk (*) denotes a significant value. Start and end positions are shown for the genomic regions that were identified to contain a shared CNV within (and across) family members. The full length of the CNV is also presented for each individual. Affection status is given for each individual, with BP-I = bipolar type I, BP-II = bipolar type II and OMD = other mental disorder. Of note, different ASMs were assumed, and affection status under a certain model implies a non-diseased status under the remaining ones. The copy number variation state is deletion or gain, where CN = 1 refers to 1 copy deletion and CN = 3 refers to 1 copy gain. All genomic coordinates are according to NCBI36/hg18. (DOC 326 kb)
