# Supplementary material for: A significant risk locus on 19q13 for bipolar disorder identified using a combined genome-wide linkage and copy number variation analysis
Source: BioData Min. 2015 Dec 18;8:42. doi: 10.1186/s13040-015-0076-y (PMC4683747; doi:10.1186/s13040-015-0076-y)
Supplement: Supplementary file 1 — Supplementary materials. (DOC 43 kb) [file 13040_2015_76_MOESM1_ESM.doc]

**Additional file 1**

**Supplementary Materials**

**Table of contents**

Study populations and ascertainment process ……………………………………………………………….............2

Quality control of individuals and SNP data ……………………………………..……..…………………………………3

CNV calling and quality control procedure of CNV data ……………..……………………………………………...4

Approach to find inherited CNVs with risk to BPD ………………………………………………………………………4

References ……………………………….……………………………………..…………………………………………………………6

**Study population and ascertainment process**

The study population was provided from the NIMH Bipolar Disorder Genetic Initiative) [1]. The ascertainment process of the bipolar spectrum disorder pedigrees (with a core phenotype of bipolar disorder type 1) was conducted from 1991 to 2001 through a screening process of clinical and non-clinical treatment facilities [2]. The pedigrees also included individuals with a diagnosis of bipolar disorder type 1 (BP-I), bipolar disorder type 2 (BP-II), schizoaffective disorder bipolar type (SABP) or recurrent unipolar disorder (RUDD). Probands were DSM-III-R diagnosed (Diagnostic and Statistical Manual of Mental Disorders, 3rd edition) for bipolar I (BP-I) and schizoaffective disorder bipolar type (SABP) or a diagnosis using RDC (Research Diagnostic Criteria) for bipolar II (BP-II) for recurrent unipolar depressive disorder (RUDD).

To ensure a high accuracy of diagnosis, multiple sources of information were systematically and carefully examined to obtain a best estimate of lifetime psychiatric diagnosis [3], [4]. These sources included the following: the DIGS (Diagnostic Interview for Genetic Studies) interview; FIGS (Family Interview for Genetic Studies) interview from all relatives who were asked about the subject, and medical records. Two senior psychiatrists or clinical psychologists, trained in these instruments, separately and independently assessed these sources of information to arrive at the best diagnosis. If disagreements remained, a third senior psychiatrist or clinical psychologist independently reviewed all sources of available information. A final best estimate diagnosis was determined by weighing all available information in a hierarchical fashion using (i) the FIGS alone (ii) the FIGS and medical records (iii) the FIGS and medical records and DIGS. Two senior psychiatrists or clinical psychologists separately and independently weighted all available information and arrived at a final best diagnosis.

Linkage analyses of the BP-pedigree Wave 1-4 samples have been published previously [5], [6], [7], [8], [9], [10]. Although several regions reached suggestive linkage in this material, no region has previously met criterion of significant linkage levels.

**Quality control procedures of individuals and SNP data**

Only SNPs with a GC score > 0.8 in the BeadStudio GenCall algorithm were included (108,006 SNPs were rejected). Initially we tested for deviation in the proportion of heterozygosity using PLINK [11] (no outliers were detected in a distributional analysis of the data). Forty-one SNPs failed test for Mendelian inheritance errors (PLINK), at ME ≥ 1, using one validated CEPH trio-sample. Filtering for poor genotype call rate (PLINK) was performed by checking distribution of the data. This approach led to exclusion of 1,436 SNPs at a genotype call rate < 0.9. Next, we re-tested the distribution and confirmed a call rate of > 99.7% in the remaining dataset. Test for duplicate errors (> 1, using duplicate genotypes from 5 individuals) led to exclusion of 947 SNPs.

Filtering for poor genotype sample call rate (PLINK) was performed by checking distribution of the data. No deviation was identified at a success rate of 98.3%. To reduce the presence of erroneous genotypes, 94,557 SNPs located within CNV-regions were zeroed out. This analysis was accomplished using CNVs identified in the CNV calling analysis described below. Informative markers for linkage analysis were selected in an independent cohort consisting of 1,000 cases and 1,000 controls from NIMH Wave 1-4 (downloaded from: http://www.ncbi.nlm.nih.gov/gap, accession number: phs000017.v3.p1) at the criterion of MAF > 0.2 and a LE with r^2^ < 0.1.

In this process 20,751 SNPs were selected. To assure correct relatedness we tested identity by descent (IBD) scores, performed in graphical relationship representation [12]. Two individuals with genotype data inconsistent with pedigree relationships were removed from the dataset. A sensitive test for identifying genotyping errors was conducted using the error detection tool in MERLIN [13]. This filter led to additional 777 markers being zeroed out using Pedwipe [13], for those families identified with a contradicting gene-flow. As a last filter we re-checked for Mendelian inconsistencies based on QC filtered data from the 46 pedigrees using the PEDSTATS software (v0.6.12) [14] and rejected 37 additional SNPs at an appearance of ME > 1.

**CNV calling and Quality control procedure of CNV data**

Raw allelic hybridization signal intensities with high quality from 598,821 polymorphic (SNP) probes and monomorphic (CNV) probes (chromosome 1-22 and X) and 275 subjects were used for CNV analysis using the PennCNV calling software [15]. The marker call rate was greater than 98%, and a call rate greater than 95% was observed for 99.4% of the DNA samples. Under these settings, 11,410 autosomal and 167 chromosome X CNVs were successfully called. A set of quality control procedures were then applied in order to reduce the false positive CNV rate. For chromosome X the pseudo-autosomal region (PAR) was removed. We detected four duplication events that involved an entire chromosome arm. As lymphoblastoid cells were used, these CNV calls were excluded from further analyses as likely cell culture artifacts. For the same reason we excluded CNVs that overlapped with the immunoglobulin regions [15]. Marker signals were required to have a standard deviation of the log R ratio (LRR) < 0.3, and CNVs were required to harbor > 10 markers and to have a genomic length between 10 kb and 10 Mb, and finally to have a confidence value > 10 (a score value that ensures a high probability of the actual integer copy number estimate being correct).

### Approach to find inherited CNVs with risk to BPD

To define genomic regions with CNVs that confer risk for BPD we developed an algorithm that calculates for linkage only in the presence of CNVs that are shared between individuals of BPD-ascertained families. The sum of average family-wise parametric LOD scores (defined as the type 1 error for one single family), or non-parametric Z scores, are calculated over regions for which families share overlapping CNVs (illustrated in Additional file 5). For families with at least two members with overlapping CNVs the average linkage score in the region is calculated and added to those observed in the same region in other families. The algorithm thus generates a CNV-weighted linkage scores for genomic segments representing regions with CNVs that are shared within and across families. This approach thus identifies inherited regions harboring CNVs that could convey risk to BPD. The CNV-weighted linkage scores are then ranked with higher score values considered to have greater impact on the phenotypic trait. Test for significance is made through a permutation analysis (label switch of case-control status) to generate null expectations of linkage data. Empirical level of significance is defined based on family-wise error rate (FWER) analysis.

Reference List

1. **NIMH Bipolar Disorder Genetic Initiative** [https://www.nimhgenetics.org/]

2. Nurnberger JI, DePaulo JR, Gershon ES, Reich T, Blehar MC, Edenberg HJ, Foroud T, Miller M, Bowman E, Mayeda A, et al: **Genomic survey of bipolar illness in the NIMH genetics initiative pedigrees: a preliminary report.** *Am J Med Genet* 1997, **74:**227-237.

3. Leckman JF, Sholomskas D, Thompson WD, Belanger A, Weissman MM: **Best estimate of lifetime psychiatric diagnosis: a methodological study.** *Arch Gen Psychiatry* 1982, **39:**879-883.

4. Weissman MM, Merikangas KR, John K, Wickramaratne P, Prusoff BA, Kidd KK: **Family-genetic studies of psychiatric disorders. Developing technologies.** *Arch Gen Psychiatry* 1986, **43:**1104-1116.

5. Edenberg HJ, Foroud T, Conneally PM, Sorbel JJ, Carr K, Crose C, Willig C, Zhao J, Miller M, Bowman E, et al: **Initial genomic scan of the NIMH genetics initiative bipolar pedigrees: chromosomes 3, 5, 15, 16, 17, and 22.** *Am J Med Genet* 1997, **74:**238-246.

6. Ross J, Berrettini W, Coryell W, Gershon ES, Badner JA, Kelsoe JR, McInnis MG, McMahon FJ, Murphy DL, Nurnberger JI, Jr., et al: **Genome-wide parametric linkage analyses of 644 bipolar pedigrees suggest susceptibility loci at chromosomes 16 and 20.** *Psychiatr Genet* 2008, **18:**191-198.

7. Detera-Wadleigh SD, Badner JA, Yoshikawa T, Sanders AR, Goldin LR, Turner G, Rollins DY, Moses T, Guroff JJ, Kazuba D, et al: **Initial genome scan of the NIMH genetics initiative bipolar pedigrees: chromosomes 4, 7, 9, 18, 19, 20, and 21q.** *Am J Med Genet* 1997, **74:**254-262.

8. Rice JP, Goate A, Williams JT, Bierut L, Dorr D, Wu W, Shears S, Gopalakrishnan G, Edenberg HJ, Foroud T, et al: **Initial genome scan of the NIMH genetics initiative bipolar pedigrees: chromosomes 1, 6, 8, 10, and 12.** *Am J Med Genet* 1997, **74:**247-253.

9. Stine OC, McMahon FJ, Chen L, Xu J, Meyers DA, MacKinnon DF, Simpson S, McInnis MG, Rice JP, Goate A, et al: **Initial genome screen for bipolar disorder in the NIMH genetics initiative pedigrees: chromosomes 2, 11, 13, 14, and X.** *Am J Med Genet* 1997, **74:**263-269.

10. Zandi PP, Willour VL, Huo Y, Chellis J, Potash JB, MacKinnon DF, Simpson SG, McMahon FJ, Gershon E, Reich T, et al: **Genome scan of a second wave of NIMH genetics initiative bipolar pedigrees: chromosomes 2, 11, 13, 14, and X.** *Am J Med Genet B Neuropsychiatr Genet* 2003, **119B:**69-76.

11. Purcell S, Neale B, Todd-Brown K, Thomas L, Ferreira MA, Bender D, Maller J, Sklar P, de Bakker PI, Daly MJ, Sham PC: **PLINK: a tool set for whole-genome association and population-based linkage analyses.** *AmJHumGenet* 2007, **81:**559-575.

12. Abecasis GR, Cherny SS, Cookson WO, Cardon LR: **GRR: graphical representation of relationship errors.** *Bioinformatics* 2001, **17:**742-743.

13. Abecasis GR, Cherny SS, Cookson WO, Cardon LR: **Merlin--rapid analysis of dense genetic maps using sparse gene flow trees.** *Nat Genet* 2002, **30:**97-101.

14. Wigginton JE, Abecasis GR: **PEDSTATS: descriptive statistics, graphics and quality assessment for gene mapping data.** *Bioinformatics* 2005, **21:**3445-3447.

15. Wang K, Li M, Hadley D, Liu R, Glessner J, Grant SF, Hakonarson H, Bucan M: **PennCNV: an integrated hidden Markov model designed for high-resolution copy number variation detection in whole-genome SNP genotyping data.** *Genome Res* 2007, **17:**1665-1674.
