# Supplementary material for: A significant risk locus on 19q13 for bipolar disorder identified using a combined genome-wide linkage and copy number variation analysis
Source: BioData Min. 2015 Dec 18;8:42. doi: 10.1186/s13040-015-0076-y (PMC4683747; doi:10.1186/s13040-015-0076-y)
Supplement: Supplementary file 2 — Selection criteria for inclusion of BP-pedigrees. Data obtained from a large number of pedigrees from NIMH Genetic Initiative Wave 1–4 was screened to generate informative pedigrees and to reduce any sporadic and environmental form of BPD. Two different analyses were used to select pedigrees. Test for runs of homozygosity (ROH) and analyzing regions with increased parametric family LOD scores under different assumed modes of inheritance. (DOC 62 kb) [file 13040_2015_76_MOESM2_ESM.doc]

| **Additional file 2.** Selection criteria for inclusion of BP-pedigrees | | | | |
| --- | --- | --- | --- | --- |
|  |  |  |  |  |
| **Pedigree-id** | **Wave** | **Selection criteria** | **Max LOD** | **Model** |
| 10-102 | 1 | LOD | 1.59 | Dominant |
| 10-114 | 1 | Multiple LODs | Several > 1.0 |  |
| 10-158 | 4 | LOD | 1.58 | Dominant |
| 10-159 | 1 | Homozygosity | NA | NA |
| 11101 | 3 | LOD | 1.53 | Recessive |
| 11-107 | 1 | LOD | 1.14 | Dominant |
| 11-114 | 1 | LOD | 1.61 | Recessive |
| 11-122 | 1 | LOD | 1.22 | Dominant |
| 11-125 | 1 | Homozygosity | NA | NA |
| 11-130 | 1 | Mendel errors | NA | NA |
| 11-150 | 1 | LOD | 2.15 | Recessive |
| 11-156 | 1 | LOD | 1.84 | Dominant |
| 11-158 | 3 | Multiple LODs | Several > 1.0 |  |
| 12-144 | 1 | Mendel errors | NA | NA |
| 12-168 | 1 | LOD | 0.96 | Dominant |
| 12-330 | 3 | Multiple LODs | Several > 1.0 |  |
| 13-101 | 1 | LOD | 1.31 | Dominant |
| 13-126 | 1 | LOD | 1.08 | Dominant |
| 20-1001 | 4 | LOD | 1.47 | Recessive |
| 20-1007 | 4 | LOD | 2.15 | Recessive |
| 20-1044 | 4 | LOD | 1.17 | Dominant |
| 20-1048 | 4 | Multiple LODs | Several > 1.0 |  |
| 20-1049 | 4 | LOD | 1.44 | Dominant |
| 21-1014 | 4 | Homozygosity | NA | NA |
| 22-1004 | 4 | Homozygosity | NA | NA |
| 23-0474 | 4 | LOD | 1.28 | Dominant |
| 24-0506 | 3 | LOD | 1 | Dominant |
| 24-0516 | 3 | LOD | 1.5 | Recessive |
| 25-1013 | 3 | Homozygosity | NA | NA |
| 25-1042 | 4 | LOD | 1.37 | Recessive |
| 25-1044 | 4 | Multiple LODs | Several > 1.0 |  |
| 26-1010 | 4 | LOD | 1.63 | Recessive |
| 26-1015 | 4 | LOD | 1.09 | Dominant |
| 26-1020 | 4 | LOD | 1.41 | Recessive |
| 26-1023 | 4 | LOD | 1.58 | Recessive |
| 26-1029 | 4 | LOD | 1.4 | Dominant |
| 26-5011 | 3 | Mendel errors | NA | NA |
| 27-1002 | 4 | LOD | 1.58 | Recessive |
| 27-1005 | 3 | Homozygosity | NA | NA |
| 28-1005 | 3 | LOD | 1.54 | Recessive |
| 29-0145 | 3 | Mendel errors | NA | NA |
| 29-0172 | 3 | Mendel errors | NA | NA |
| 29-0174 | 3 | LOD | 1.53 | Recessive |
| 29-0209 | 3 | Mendel errors | NA | NA |
| 29-0250 | 3 | LOD | 1.57 | Recessive |
| 29-0254 | 4 | Homozygosity | NA | NA |
