# Supplementary figures and images for: A significant risk locus on 19q13 for bipolar disorder identified using a combined genome-wide linkage and copy number variation analysis
Source: BioData Min. 2015 Dec 18;8:42. doi: 10.1186/s13040-015-0076-y (PMC4683747; doi:10.1186/s13040-015-0076-y)

Additional file 3:

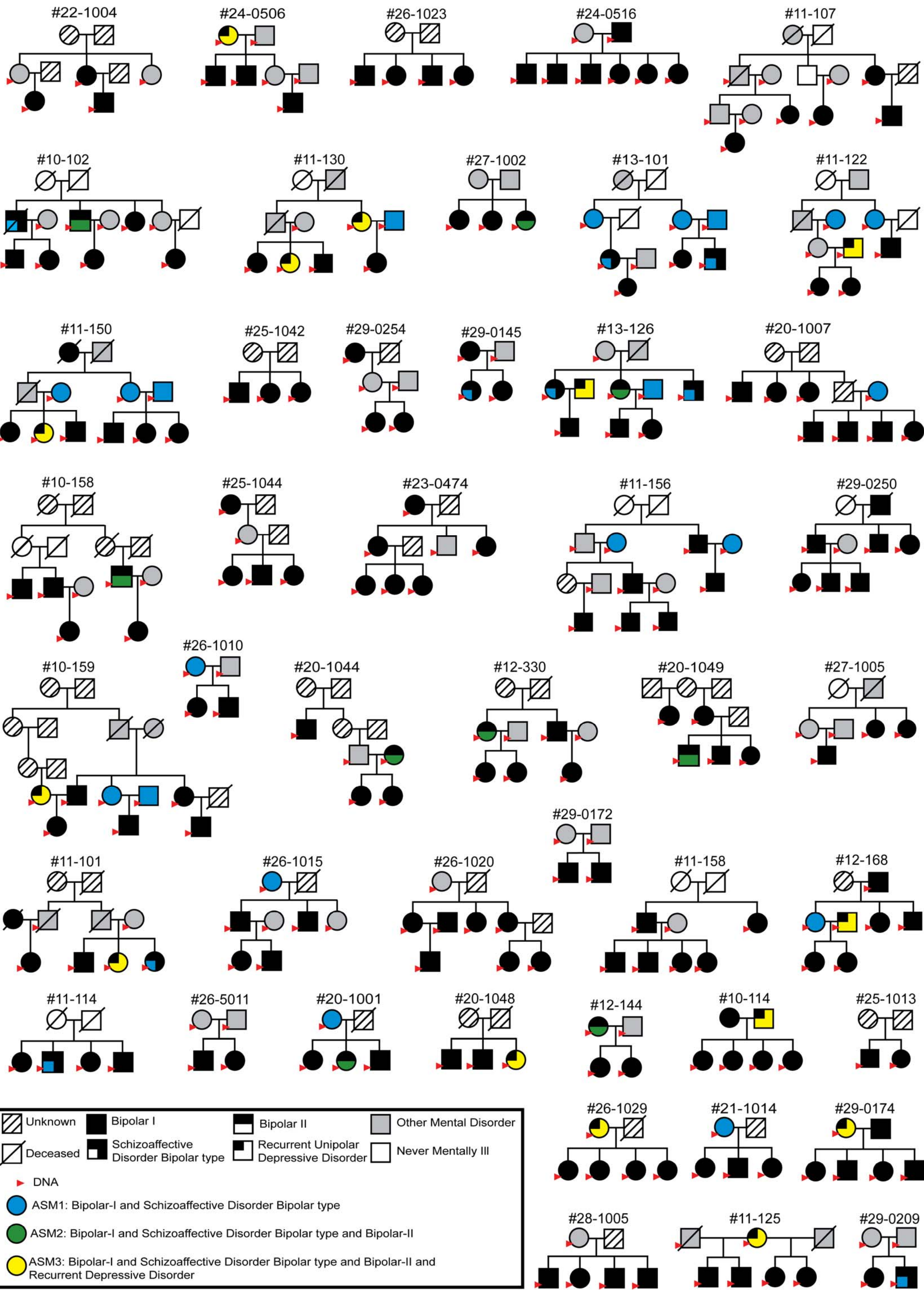

Supplement: Supplementary file 3 — Pedigrees included in linkage and CNV weighted analysis for BPD. To retrieve informative pedigrees from the NIMH Genetic Initiative Wave 1–4 sample, 46 pedigrees consisting of 269 genotyped individuals and 97 individuals with no available DNA were selected. Three affection status models were considered (ASM1-3) based on the different bipolar affective disorder sub-types, described in the figure-box. Individuals with a diagnosis of bipolar spectrum disorders which only apply to a certain ASM were coded as “unknown” under the other ASMs. In order to illustrate full details of pedigree composition different subtypes of bipolar spectrum disorders are illustrated. For linkage analyses with different ASMs only genotyped individuals were coded as ASM1-3. All other individuals, irrespective of diagnosis, were coded as ‘unknown’. (PDF 364 kb) [file 13040_2015_76_MOESM3_ESM.pdf]

CNV-weighted linkage score  
(Region with 3 markers)

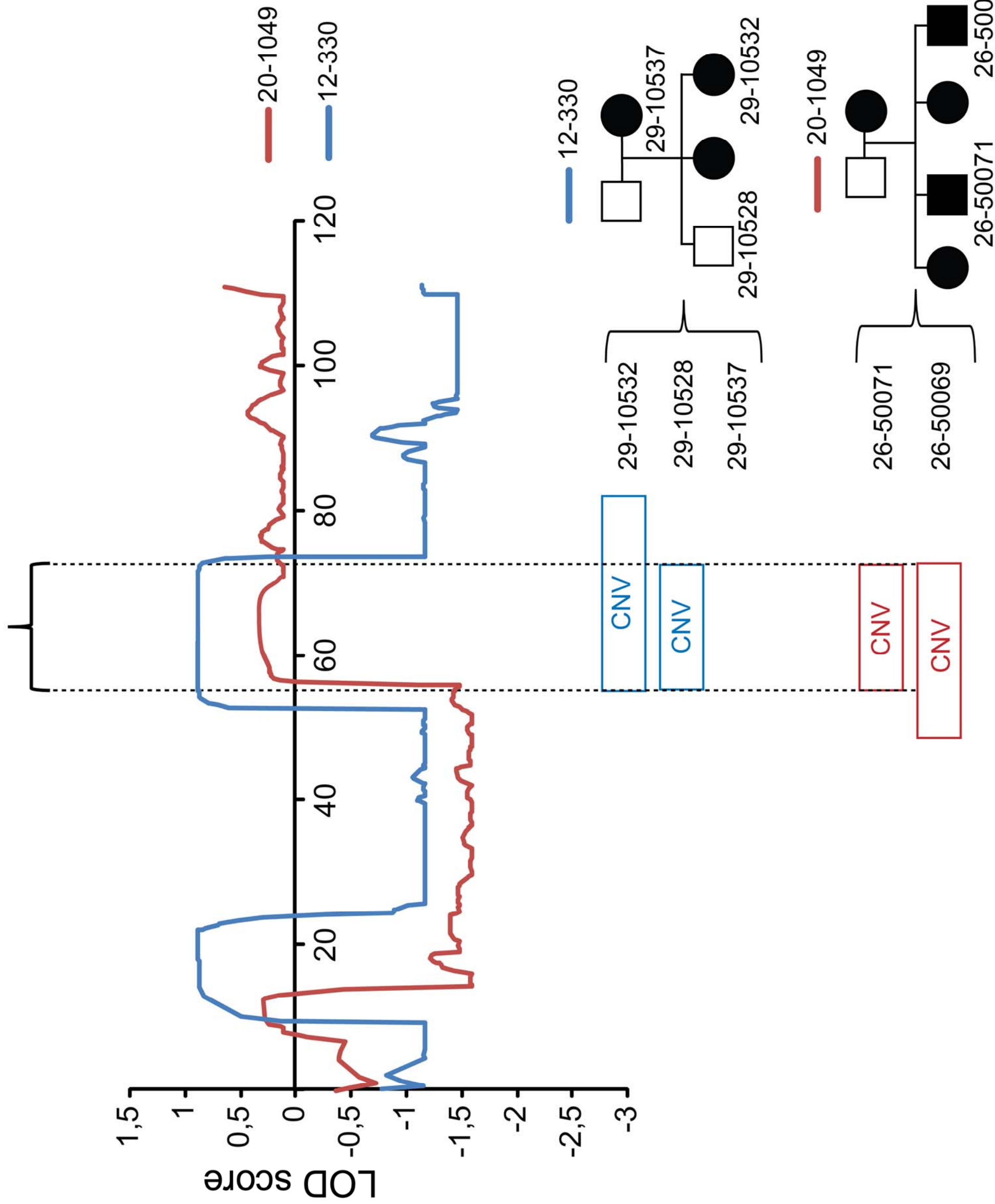

Supplement: Supplementary file 5 — Principles for region for which one CNV-weighted linkage score is calculated. LOD scores from two families (family ID: 20–1049 and 12–330) for a certain chromosome are depicted. The occurrence of CNVs for individuals within these families is also illustrated. The average linkage score (parametric LOD score or non-parametric Z-score) is calculated over the region with overlapping CNV from at least two individuals in the same family. Note, for those individuals with the presence of a CNV were SNPs zeroed out. In the non-CNV carriers were genotypes intact. The average linkage score from all families with overlapping CNV in at least two individuals is added. A CNV-weighted linkage score is thus generated for a defined region that share overlapping CNVs for more than 1 individual per family. To illustrate the calculation; an example is given with three markers that are located in a region with four overlapping CNVs, at the position 60–75 cM. For these 3 markers, the LOD scores for the two families are: Fam-ID 12–330: 0.9, 0.9 and 0.9, Fam-ID: 20–1049: 0.2, 0.4 and 0.1. The CNV-weighted linkage score (the sum of average linkage score) is then calculated as: Fam-ID 12–330: (0.9 + 0.9 + 0.9)/3 = 0.9, Fam-ID: 20–1049: (0.2 + 0.4 + 0.1)/3 = 0.23. Then, a total CNV-weighted linkage score = 0.9 + 0.23 = 1.13. (PDF 179 kb) [file 13040_2015_76_MOESM5_ESM.pdf]
