# Supplementary material for: A significant risk locus on 19q13 for bipolar disorder identified using a combined genome-wide linkage and copy number variation analysis
Source: BioData Min. 2015 Dec 18;8:42. doi: 10.1186/s13040-015-0076-y (PMC4683747; doi:10.1186/s13040-015-0076-y)
Supplement: Supplementary file 4 — Results of the parametric and non-parametric genome-wide linkage analyses under affection status models ASM1-3. Simulation based thresholds for genome-wide significant and suggestive linkage levels are illustrated in red and black dashed lines respectively. A-C: Non-parametric linkage analyses. NPL Z scores are illustrated. D-F: Parametric dominant linkage analyses. Heterogeneity LOD scores (HLOD) are illustrated. G-I: Parametric recessive linkage analyses. Heterogeneity LOD scores (HLOD) are illustrated. (PDF 367 kb) [file 13040_2015_76_MOESM4_ESM.pdf]

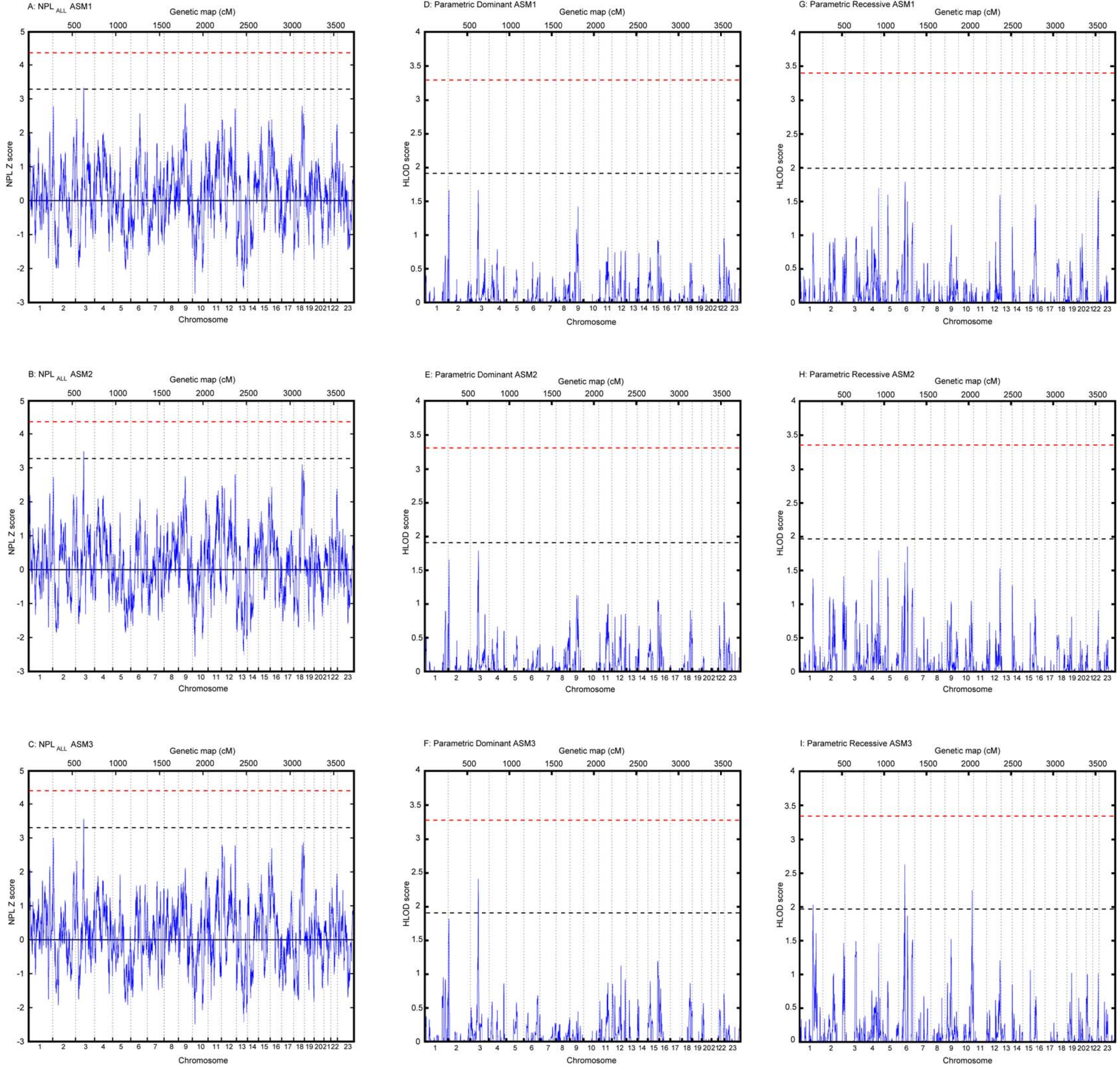
A: NPL ALL ASM1
B: NPL ALL ASM2
C: NPL ALL ASM3
D: Parametric Dominant ASM1
E: Parametric Dominant ASM2
F: Parametric Dominant ASM3
G: Parametric Recessive ASM1
H: Parametric Recessive ASM2
I: Parametric Recessive ASM3
Genetic map (cM)
Chromosome
NPL Z score
HLOD score
