# Supplementary material for: A significant risk locus on 19q13 for bipolar disorder identified using a combined genome-wide linkage and copy number variation analysis
Source: BioData Min. 2015 Dec 18;8:42. doi: 10.1186/s13040-015-0076-y (PMC4683747; doi:10.1186/s13040-015-0076-y)
Supplement: Supplementary file 6 — Genomic positions of CNVs identified in 19q13. The table shows the positions of all CNVs that were identified in 19q13 (chr19:48066441–48157656) and that harbors a significant CNV-weighted linkage score. All genomic coordinates are according to NCBI36/hg18. (DOC 38 kb) [file 13040_2015_76_MOESM6_ESM.doc]

| **Additional file 6. G**enomic positions of CNVs identified in 19q13. | | | | |
| --- | --- | --- | --- | --- |
|  | **Start position (bp)** | **End position (bp)** | **Pedigree id** | **Individ id** |
| **Chr** |
| Chr19 | 48009908 | 48160500 | 11-130 | 11-11113 |
| Chr19 | 48013905 | 48160500 | 11-156 | 11-12163 |
| Chr19 | 48009908 | 48418735 | 12-330 | 12-11239 |
| Chr19 | 47982103 | 48430180 | 12-330 | 12-11240 |
| Chr19 | 48009908 | 48114839 | 12-330 | 12-11241 |
| Chr19 | 48009908 | 48205499 | 20-1049 | 20-10856 |
| Chr19 | 47948855 | 48387680 | 20-1049 | 20-10868 |
| chr19 | 47997996 | 48166539 | 26-5011 | 26-50069 |
| Chr19 | 47997996 | 48205499 | 26-5011 | 26-50071 |
| Chr19 | 48066441 | 48114839 | 29-0172 | 29-10511 |
| Chr19 | 46046373 | 46064315 | 29-0172 | 29-10514 |
| Chr19 | 47997996 | 48205499 | 29-0174 | 29-10528 |
| Chr19 | 47997996 | 48160500 | 29-0174 | 29-10532 |
| Chr19 | 47997996 | 48160500 | 29-0174 | 29-10535 |
| Chr19 | 48066441 | 48157656 | 29-0209 | 29-10642 |
| Chr19 | 48066441 | 48205499 | 29-0209 | 29-10656 |
