# Supplementary material for: A significant risk locus on 19q13 for bipolar disorder identified using a combined genome-wide linkage and copy number variation analysis
Source: BioData Min. 2015 Dec 18;8:42. doi: 10.1186/s13040-015-0076-y (PMC4683747; doi:10.1186/s13040-015-0076-y)
Supplement: Supplementary file 7 — Table of the 25 strongest CNV-weighted linkage scores of the parametric dominant ASM1 model. The two strongest signals withstood test for significance using a 1000-fold simulation analysis including a FWER correction. Asterisk (*) denotes a significant value. Start and end positions are shown for the genomic regions that were identified to contain a shared CNV within (and across) family members. The full length of the CNV is also presented for each individual. Affection status is given for each individual, with BP-I = bipolar type I, BP-II = bipolar type II and OMD = other mental disorder. Of note, different ASMs were assumed, and affection status under a certain model implies a non-diseased status under the remaining ones. The copy number variation state is deletion or gain, where CN = 1 refers to 1 copy deletion and CN = 3 refers to 1 copy gain. All genomic coordinates are according to NCBI36/hg18. (DOC 326 kb) [file 13040_2015_76_MOESM7_ESM.doc]

| **Additional file 7.** Table of the 25 strongest CNV-weighted linkage scores of the parametric dominant ASM1 model | | | | | | | | | | | |
| --- | --- | --- | --- | --- | --- | --- | --- | --- | --- | --- | --- |
|  |  |  |  |  |  |  |  |  |  |  |  |
|  | **Statistics of CNV-weighted linkage scores** | **Genomic region calculated with CNV-weighted linkage analysis** | |  |  |  |  |  |  |  |  |
|  |  |  |  |  |  |  |  |  |  |  |  |
|  |  |  |  |  | **Genomic region with** | |  |  |  |  |  |
|  |  |  |  |  | **CNV** | | **Pedigree id** | **Individ id** | **Affection status** |  |  |
| **Chr** |  | **Start (bp)** | **End (bp)** |  | **Start (bp)** | **End (bp)** |  |  |  | **Ethnicity** | **CN state** |
| 19 | 2.578* | 48066441 | 48114839 |  | 48066441 | 48157656 | 29-0209 | 29-10642 | BP-I | White | 1 |
|  |  |  |  |  | 48066441 | 48205499 | 29-0209 | 29-10656 | OMD | White | 1 |
|  |  |  |  |  | 47997996 | 48160500 | 29-0174 | 29-10532 | BP-I | White | 1 |
|  |  |  |  |  | 47997996 | 48160500 | 29-0174 | 29-10535 | BP-I | White | 1 |
|  |  |  |  |  | 47997996 | 48205499 | 29-0174 | 29-10528 | BP-I | White | 1 |
|  |  |  |  |  | 47997996 | 48166539 | 26-5011 | 26-50069 | BP-I | White | 1 |
|  |  |  |  |  | 47997996 | 48205499 | 26-5011 | 26-50071 | OMD | White | 1 |
|  |  |  |  |  | 47948855 | 48387680 | 20-1049 | 20-10868 | BP-II (asm2) | White | 3 |
|  |  |  |  |  | 48009908 | 48205499 | 20-1049 | 20-10856 | BP-I | White | 3 |
|  |  |  |  |  | 47982103 | 48430180 | 12-330 | 12-11240 | BP-I | White | 1 |
|  |  |  |  |  | 48009908 | 48114839 | 12-330 | 12-11241 | BP-I | White | 1 |
|  |  |  |  |  | 48009908 | 48418735 | 12-330 | 12-11239 | OMD | White | 1 |
|  |  |  |  |  |  |  |  |  |  |  |  |
|  |  |  |  |  |  |  |  |  |  |  |  |
| 19 | 2.577* | 48114839 | 48157656 |  | 48066441 | 48157656 | 29-0209 | 29-10642 | BP-I | White | 1 |
|  |  |  |  |  | 48066441 | 48205499 | 29-0209 | 29-10656 | OMD | White | 1 |
|  |  |  |  |  | 47997996 | 48160500 | 29-0174 | 29-10532 | BP-I | White | 1 |
|  |  |  |  |  | 47997996 | 48160500 | 29-0174 | 29-10535 | BP-I | White | 1 |
|  |  |  |  |  | 47997996 | 48205499 | 29-0174 | 29-10528 | BP-I | White | 1 |
|  |  |  |  |  | 47997996 | 48166539 | 26-5011 | 26-50069 | BP-I | White | 1 |
|  |  |  |  |  | 47997996 | 48205499 | 26-5011 | 26-50071 | OMD | White | 1 |
|  |  |  |  |  | 47948855 | 48387680 | 20-1049 | 20-10868 | BP-II (asm2) | White | 3 |
|  |  |  |  |  | 48009908 | 48205499 | 20-1049 | 20-10856 | BP-I | White | 3 |
|  |  |  |  |  | 47982103 | 48430180 | 12-330 | 12-11240 | BP-I | White | 1 |
|  |  |  |  |  | 48009908 | 48418735 | 12-330 | 12-11239 | OMD | White | 1 |
|  |  |  |  |  |  |  |  |  |  |  |  |
|  |  |  |  |  |  |  |  |  |  |  |  |
| 19 | 2.285 | 48009908 | 48013905 |  | 47997996 | 48160500 | 29-0174 | 29-10532 | BP-II | White | 1 |
|  |  |  |  |  | 47997996 | 48160500 | 29-0174 | 29-10535 | BP-II | White | 1 |
|  |  |  |  |  | 47997996 | 48205499 | 29-0174 | 29-10528 | BP-II | White | 1 |
|  |  |  |  |  | 47997996 | 48166539 | 26-5011 | 26-50069 | BP-II | Black | 1 |
|  |  |  |  |  | 47997996 | 48205499 | 26-5011 | 26-50071 | OMD | Black | 1 |
|  |  |  |  |  | 47948855 | 48387680 | 20-1049 | 20-10868 | BP-II (asm2) | White | 3 |
|  |  |  |  |  | 48009908 | 48205499 | 20-1049 | 20-10856 | BP-II | White | 3 |
|  |  |  |  |  | 47982103 | 48430180 | 12-330 | 12-11240 | BP-II | White | 1 |
|  |  |  |  |  | 48009908 | 48114839 | 12-330 | 12-11241 | BP-II | White | 1 |
|  |  |  |  |  | 48009908 | 48418735 | 12-330 | 12-11239 | BP-II | White | 1 |
|  |  |  |  |  |  |  |  |  |  |  |  |
|  |  |  |  |  |  |  |  |  |  |  |  |
| 19 | 2.284 | 48013905 | 48066441 |  | 47997996 | 48160500 | 29-0174 | 29-10532 | BP-II | White | 1 |
|  |  |  |  |  | 47997996 | 48160500 | 29-0174 | 29-10535 | BP-II | White | 1 |
|  |  |  |  |  | 47997996 | 48205499 | 29-0174 | 29-10528 | BP-II | White | 1 |
|  |  |  |  |  | 47997996 | 48166539 | 26-5011 | 26-50069 | BP-II | Black | 1 |
|  |  |  |  |  | 47997996 | 48205499 | 26-5011 | 26-50071 | OMD | Black | 1 |
|  |  |  |  |  | 47948855 | 48387680 | 20-1049 | 20-10868 | BP-II (asm2) | White | 3 |
|  |  |  |  |  | 48009908 | 48205499 | 20-1049 | 20-10856 | BP-II | White | 3 |
|  |  |  |  |  | 47982103 | 48430180 | 12-330 | 12-11240 | BP-II | White | 1 |
|  |  |  |  |  | 48009908 | 48114839 | 12-330 | 12-11241 | BP-II | White | 1 |
|  |  |  |  |  | 48009908 | 48418735 | 12-330 | 12-11239 | BP-II | White | 1 |
|  |  |  |  |  |  |  |  |  |  |  |  |
|  |  |  |  |  |  |  |  |  |  |  |  |
| 19 | 2.282 | 48157656 | 48160500 |  | 47997996 | 48160500 | 29-0174 | 29-10532 | BP-II | White | 1 |
|  |  |  |  |  | 47997996 | 48160500 | 29-0174 | 29-10535 | BP-II | White | 1 |
|  |  |  |  |  | 47997996 | 48205499 | 29-0174 | 29-10528 | BP-II | White | 1 |
|  |  |  |  |  | 47997996 | 48166539 | 26-5011 | 26-50069 | BP-II | Black | 1 |
|  |  |  |  |  | 47997996 | 48205499 | 26-5011 | 26-50071 | OMD | Black | 1 |
|  |  |  |  |  | 47948855 | 48387680 | 20-1049 | 20-10868 | BP-II (asm2) | White | 3 |
|  |  |  |  |  | 48009908 | 48205499 | 20-1049 | 20-10856 | BP-II | White | 3 |
|  |  |  |  |  | 47982103 | 48430180 | 12-330 | 12-11240 | BP-II | White | 1 |
|  |  |  |  |  | 48009908 | 48418735 | 12-330 | 12-11239 | BP-II | White | 1 |
|  |  |  |  |  |  |  |  |  |  |  |  |
|  |  |  |  |  |  |  |  |  |  |  |  |
| 12 | 1.745 | 62374612 | 62405452 |  | 62226007 | 62405452 | 11-107 | 11-10033 | OMD | White | 3 |
|  |  |  |  |  | 62226007 | 62405452 | 11-107 | 11-10054 | BP-II | White | 3 |
|  |  |  |  |  | 62233961 | 62405452 | 11-107 | 11-10029 | BP-II | White | 3 |
|  |  |  |  |  | 62233961 | 62405452 | 11-107 | 11-10112 | BP-II | White | 3 |
|  |  |  |  |  | 62233961 | 62405452 | 11-107 | 11-10242 | BP-II | White | 3 |
|  |  |  |  |  |  |  |  |  |  |  |  |
|  |  |  |  |  |  |  |  |  |  |  |  |
| 12 | 1.745 | 62347652 | 62374612 |  | 62218146 | 62374612 | 11-107 | 11-10213 | OMD | White | 3 |
|  |  |  |  |  | 62226007 | 62405452 | 11-107 | 11-10033 | OMD | White | 3 |
|  |  |  |  |  | 62226007 | 62405452 | 11-107 | 11-10054 | BP-II | White | 3 |
|  |  |  |  |  | 62233961 | 62405452 | 11-107 | 11-10029 | BP-II | White | 3 |
|  |  |  |  |  | 62233961 | 62405452 | 11-107 | 11-10112 | BP-II | White | 3 |
|  |  |  |  |  | 62233961 | 62405452 | 11-107 | 11-10242 | BP-II | White | 3 |
|  |  |  |  |  |  |  |  |  |  |  |  |
|  |  |  |  |  |  |  |  |  |  |  |  |
| 12 | 1.745 | 62338144 | 62347652 |  | 62218146 | 62374612 | 11-107 | 11-10213 | OMD | White | 3 |
|  |  |  |  |  | 62226007 | 62405452 | 11-107 | 11-10033 | OMD | White | 3 |
|  |  |  |  |  | 62226007 | 62405452 | 11-107 | 11-10054 | BP-II | White | 3 |
|  |  |  |  |  | 62233961 | 62405452 | 11-107 | 11-10029 | BP-II | White | 3 |
|  |  |  |  |  | 62233961 | 62405452 | 11-107 | 11-10112 | BP-II | White | 3 |
|  |  |  |  |  | 62233961 | 62405452 | 11-107 | 11-10242 | BP-II | White | 3 |
|  |  |  |  |  |  |  |  |  |  |  |  |
|  |  |  |  |  |  |  |  |  |  |  |  |
| 12 | 1.745 | 62233961 | 62249712 |  | 62218146 | 62374612 | 11-107 | 11-10213 | OMD | White | 3 |
|  |  |  |  |  | 62226007 | 62405452 | 11-107 | 11-10033 | OMD | White | 3 |
|  |  |  |  |  | 62226007 | 62405452 | 11-107 | 11-10054 | BP-II | White | 3 |
|  |  |  |  |  | 62233961 | 62405452 | 11-107 | 11-10029 | BP-II | White | 3 |
|  |  |  |  |  | 62233961 | 62405452 | 11-107 | 11-10112 | BP-II | White | 3 |
|  |  |  |  |  | 62233961 | 62405452 | 11-107 | 11-10242 | BP-II | White | 3 |
|  |  |  |  |  |  |  |  |  |  |  |  |
|  |  |  |  |  |  |  |  |  |  |  |  |
| 12 | 1.745 | 62226007 | 62233961 |  | 62218146 | 62374612 | 11-107 | 11-10213 | OMD | White | 3 |
|  |  |  |  |  | 62226007 | 62405452 | 11-107 | 11-10033 | OMD | White | 3 |
|  |  |  |  |  | 62226007 | 62405452 | 11-107 | 11-10054 | BP-II | White | 3 |
|  |  |  |  |  |  |  |  |  |  |  |  |
| 12 | 1.745 | 19360345 | 19455989 |  | 19360345 | 19455989 | 11-107 | 11-10033 | OMD | White | 3 |
|  |  |  |  |  | 19358223 | 19455989 | 11-107 | 11-10054 | BP-II | White | 3 |
|  |  |  |  |  |  |  |  |  |  |  |  |
|  |  |  |  |  |  |  |  |  |  |  |  |
| 19 | 1.744 | 48531928 | 48537433 |  | 48354926 | 48537433 | 20-1044 | 20-10790 | Unknown | White | 1 |
|  |  |  |  |  | 48457670 | 48537433 | 20-1044 | 20-10788 | BP-II | White | 1 |
|  |  |  |  |  | 48354926 | 48537433 | 12-330 | 12-11232 | BP-II (asm2) | White | 1 |
|  |  |  |  |  | 48358537 | 48537433 | 12-330 | 12-11230 | BP-II | White | 1 |
|  |  |  |  |  |  |  |  |  |  |  |  |
|  |  |  |  |  |  |  |  |  |  |  |  |
| 19 | 1.703 | 48160500 | 48166539 |  | 47997996 | 48166539 | 26-5011 | 26-50069 | BP-II | Black | 1 |
|  |  |  |  |  | 47997996 | 48205499 | 26-5011 | 26-50071 | OMD | Black | 1 |
|  |  |  |  |  | 47948855 | 48387680 | 20-1049 | 20-10868 | BP-II (asm2) | White | 3 |
|  |  |  |  |  | 48009908 | 48205499 | 20-1049 | 20-10856 | BP-II | White | 3 |
|  |  |  |  |  | 47982103 | 48430180 | 12-330 | 12-11240 | BP-II | White | 1 |
|  |  |  |  |  | 48009908 | 48418735 | 12-330 | 12-11239 | OMD | White | 1 |
|  |  |  |  |  |  |  |  |  |  |  |  |
|  |  |  |  |  |  |  |  |  |  |  |  |
| 19 | 1.703 | 48166539 | 48184865 |  | 47948855 | 48387680 | 20-1049 | 20-10868 | BP-II (asm2) | White | 3 |
|  |  |  |  |  | 48009908 | 48205499 | 20-1049 | 20-10856 | BP-II | White | 3 |
|  |  |  |  |  | 47982103 | 48430180 | 12-330 | 12-11240 | BP-II | White | 1 |
|  |  |  |  |  | 48009908 | 48418735 | 12-330 | 12-11239 | OMD | White | 1 |
|  |  |  |  |  |  |  |  |  |  |  |  |
|  |  |  |  |  |  |  |  |  |  |  |  |
| 19 | 1.703 | 48184865 | 48205499 |  | 47948855 | 48387680 | 20-1049 | 20-10868 | BP-II (asm2) | White | 3 |
|  |  |  |  |  | 48009908 | 48205499 | 20-1049 | 20-10856 | BP-II | White | 3 |
|  |  |  |  |  | 47982103 | 48430180 | 12-330 | 12-11240 | BP-II | White | 1 |
|  |  |  |  |  | 48009908 | 48418735 | 12-330 | 12-11239 | OMD | White | 1 |
|  |  |  |  |  | 48184865 | 48387680 | 12-330 | 12-11241 | BP-II | White | 1 |
|  |  |  |  |  |  |  |  |  |  |  |  |
|  |  |  |  |  |  |  |  |  |  |  |  |
| 19 | 1.700 | 48333776 | 48338970 |  | 47948855 | 48387680 | 20-1049 | 20-10868 | BP-II (asm2) | White | 3 |
|  |  |  |  |  | 48333776 | 48387680 | 20-1049 | 20-10856 | BP-II | White | 3 |
|  |  |  |  |  | 47982103 | 48430180 | 12-330 | 12-11240 | BP-II | White | 1 |
|  |  |  |  |  | 48009908 | 48418735 | 12-330 | 12-11239 | OMD | White | 1 |
|  |  |  |  |  | 48184865 | 48387680 | 12-330 | 12-11241 | BP-II | White | 1 |
|  |  |  |  |  |  |  |  |  |  |  |  |
|  |  |  |  |  |  |  |  |  |  |  |  |
| 19 | 1.699 | 48338970 | 48354926 |  | 47948855 | 48387680 | 20-1049 | 20-10868 | BP-II (asm2) | White | 3 |
|  |  |  |  |  | 48333776 | 48387680 | 20-1049 | 20-10856 | BP-II | White | 3 |
|  |  |  |  |  | 47982103 | 48430180 | 12-330 | 12-11240 | BP-II | White | 1 |
|  |  |  |  |  | 48009908 | 48418735 | 12-330 | 12-11239 | OMD | White | 1 |
|  |  |  |  |  | 48184865 | 48387680 | 12-330 | 12-11241 | BP-II | White | 1 |
|  |  |  |  |  |  |  |  |  |  |  |  |
|  |  |  |  |  |  |  |  |  |  |  |  |
| 19 | 1.699 | 48354926 | 48358537 |  | 47948855 | 48387680 | 20-1049 | 20-10868 | BP-II (asm2) | White | 3 |
|  |  |  |  |  | 48333776 | 48387680 | 20-1049 | 20-10856 | BP-II | White | 3 |
|  |  |  |  |  | 47982103 | 48430180 | 12-330 | 12-11240 | BP-II | White | 1 |
|  |  |  |  |  | 48009908 | 48418735 | 12-330 | 12-11239 | OMD | White | 1 |
|  |  |  |  |  | 48184865 | 48387680 | 12-330 | 12-11241 | BP-II | White | 1 |
|  |  |  |  |  | 48354926 | 48537433 | 12-330 | 12-11232 | BP-II (asm2) | White | 1 |
|  |  |  |  |  |  |  |  |  |  |  |  |
|  |  |  |  |  |  |  |  |  |  |  |  |
| 19 | 1.699 | 48358537 | 48387680 |  | 47948855 | 48387680 | 20-1049 | 20-10868 | BP-II (asm2) | White | 3 |
|  |  |  |  |  | 48333776 | 48387680 | 20-1049 | 20-10856 | BP-II | White | 3 |
|  |  |  |  |  | 47982103 | 48430180 | 12-330 | 12-11240 | BP-II | White | 1 |
|  |  |  |  |  | 48009908 | 48418735 | 12-330 | 12-11239 | OMD | White | 1 |
|  |  |  |  |  | 48184865 | 48387680 | 12-330 | 12-11241 | BP-II | White | 1 |
|  |  |  |  |  | 48354926 | 48537433 | 12-330 | 12-11232 | BP-II (asm2) | White | 1 |
|  |  |  |  |  | 48358537 | 48537433 | 12-330 | 12-11230 | BP-II | White | 1 |
|  |  |  |  |  |  |  |  |  |  |  |  |
|  |  |  |  |  |  |  |  |  |  |  |  |
| 3 | 1.311 | 174722147 | 174730836 |  | 174722147 | 174783731 | 24-0516 | 24-10216 | BP-II | White | 3 |
|  |  |  |  |  | 174722147 | 174783731 | 24-0516 | 24-10219 | BP-II | White | 3 |
|  |  |  |  |  | 174722147 | 174783731 | 11-150 | 11-12023 | BP-II | White | 3 |
|  |  |  |  |  | 174722147 | 174783731 | 11-150 | 11-12022 | BP-II | White | 3 |
|  |  |  |  |  | 174722147 | 174783731 | 11-150 | 11-12012 | BP-II (asm1) | White | 3 |
|  |  |  |  |  | 174716048 | 174771975 | 10-159 | 10-10699 | BP-II | White | 3 |
|  |  |  |  |  | 174716048 | 174771975 | 10-159 | 10-10708 | BP-II | White | 3 |
|  |  |  |  |  | 174722147 | 174771975 | 10-159 | 10-10710 | BP-II (asm1) | White | 3 |
|  |  |  |  |  | 174722147 | 174771975 | 10-159 | 10-10730 | BP-II | White | 3 |
|  |  |  |  |  |  |  |  |  |  |  |  |
|  |  |  |  |  |  |  |  |  |  |  |  |
| 12 | 1.210 | 62249712 | 62338144 |  | 62218146 | 62374612 | 11-107 | 11-10213 | OMD | White | 3 |
|  |  |  |  |  | 62226007 | 62405452 | 11-107 | 11-10033 | OMD | White | 3 |
|  |  |  |  |  | 62226007 | 62405452 | 11-107 | 11-10054 | BP-II | White | 3 |
|  |  |  |  |  | 62233961 | 62405452 | 11-107 | 11-10029 | BP-II | White | 3 |
|  |  |  |  |  | 62233961 | 62405452 | 11-107 | 11-10112 | BP-II | White | 3 |
|  |  |  |  |  | 62233961 | 62405452 | 11-107 | 11-10242 | BP-II | White | 3 |
|  |  |  |  |  |  |  |  |  |  |  |  |
|  |  |  |  |  |  |  |  |  |  |  |  |
| 3 | 1.137 | 65649762 | 65844632 |  | 65649762 | 65844632 | 11-158 | 11-12296 | BP-II | White | 1 |
|  |  |  |  |  | 65649762 | 65844632 | 11-158 | 11-12293 | BP-II | White | 1 |
|  |  |  |  |  | 65649762 | 65844632 | 11-158 | 11-12290 | BP-II | White | 1 |
|  |  |  |  |  | 65649762 | 65848146 | 11-158 | 11-12288 | BP-II | White | 1 |
|  |  |  |  |  | 65649762 | 65848146 | 11-158 | 11-12295 | BP-II | White | 1 |
|  |  |  |  |  | 65649762 | 65848146 | 11-158 | 11-12287 | BP-II | White | 1 |
|  |  |  |  |  |  |  |  |  |  |  |  |
|  |  |  |  |  |  |  |  |  |  |  |  |
| 3 | 1.137 | 65844632 | 65848146 |  | 65649762 | 65848146 | 11-158 | 11-12288 | BP-II | White | 1 |
|  |  |  |  |  | 65649762 | 65848146 | 11-158 | 11-12295 | BP-II | White | 1 |
|  |  |  |  |  | 65649762 | 65848146 | 11-158 | 11-12287 | BP-II | White | 1 |
|  |  |  |  |  |  |  |  |  |  |  |  |
|  |  |  |  |  |  |  |  |  |  |  |  |
| 3 | 0.938 | 174771975 | 174783731 |  | 174722147 | 174783731 | 24-0516 | 24-10216 | BP-II | White | 3 |
|  |  |  |  |  | 174722147 | 174783731 | 24-0516 | 24-10219 | BP-II | White | 3 |
|  |  |  |  |  | 174722147 | 174783731 | 11-150 | 11-12023 | BP-II | White | 3 |
|  |  |  |  |  | 174722147 | 174783731 | 11-150 | 11-12022 | BP-II | White | 3 |
|  |  |  |  |  | 174722147 | 174783731 | 11-150 | 11-12012 | BP-II (asm1) | White | 3 |
|  |  |  |  |  |  |  |  |  |  |  |  |
| 19 | 0.878 | 48205499 | 48333776 |  | 47982103 | 48430180 | 12-330 | 12-11240 | BP-II | White | 1 |
|  |  |  |  |  | 48009908 | 48418735 | 12-330 | 12-11239 | BP-II | White | 1 |
|  |  |  |  |  | 48184865 | 48387680 | 12-330 | 12-11241 | BP-II | White | 1 |
